# Flapping vibrations contributed to heat insulation in Cs_2_ZnI_4_

**DOI:** 10.1093/nsr/nwag256

**Published:** 2026-05-01

**Authors:** Linjie Wu, Pengfei Nan, Long Yang, Zhiwei Chen, Changyuan Li, Qingyu Bai, Hongzheng Wang, Sui Ting Tai, Chen Wang, Binghui Ge, Yue Chen, Jun Luo, Yanzhong Pei

**Affiliations:** Interdisciplinary Materials Research Center, School of Materials Science and Engineering, Tongji University, Shanghai 201804, China; Institutes of Physical Science and Information Technology, Anhui University, Hefei 230601, China; Interdisciplinary Materials Research Center, School of Materials Science and Engineering, Tongji University, Shanghai 201804, China; Interdisciplinary Materials Research Center, School of Materials Science and Engineering, Tongji University, Shanghai 201804, China; Interdisciplinary Materials Research Center, School of Materials Science and Engineering, Tongji University, Shanghai 201804, China; Interdisciplinary Materials Research Center, School of Materials Science and Engineering, Tongji University, Shanghai 201804, China; Institutes of Physical Science and Information Technology, Anhui University, Hefei 230601, China; Department of Mechanical Engineering, The University of Hong Kong, Hong Kong 999077, China; Department of Mechanical Engineering, The University of Hong Kong, Hong Kong 999077, China; Institutes of Physical Science and Information Technology, Anhui University, Hefei 230601, China; Department of Mechanical Engineering, The University of Hong Kong, Hong Kong 999077, China; Interdisciplinary Materials Research Center, School of Materials Science and Engineering, Tongji University, Shanghai 201804, China; Interdisciplinary Materials Research Center, School of Materials Science and Engineering, Tongji University, Shanghai 201804, China

**Keywords:** flapping vibrations, single crystal, heat insulation

## Abstract

Shear modulus is commonly lower than its elastic counterpart in solids, leading to our experience that in the elastic region, it is usually much easier to shear than to compress/stretch a solid. Scientifically, this implies an easier change in molecular bonding angle than bonding length, further corresponding to the normally observed phenomenon that the propagation of longitudinal lattice vibrations is faster than that of shear ones. Populating the easier vibrations of flapping bond angles fundamentally opens an opportunity to insulate heat conduction in solids, since this type of vibration ensures slow propagation and low energy/frequency, leading to a low thermal conductivity. Here we demonstrate that single-crystalline orthorhombic Cs_2_ZnI_4_, with two sets of extremely low-frequency flapping vibrations in the crystallographic planes of *ac* (0.4 THz) and *bc* (0.5 THz), respectively, exhibits extraordinarily low thermal conductivities of 0.11 W m^−1^ K^−1^ along the *c* direction and of 0.16 W m^−1^ K^−1^ along the *b* direction at room temperature, as compared to ∼0.2 W m^−1^ K^−1^ in existing heat insulators in the dense form. The strategy and material developed in this work are believed to enrich the advancements of heat insulation technology.

## Teaser

Flapping vibrations at an extremely low frequency of 0.4 THz give single-crystal Cs_2_ZnI_4_ an ultra-low thermal conductivity of 0.11 W m^−1^ K^−1^ along its *c*-axis.

## INTRODUCTION

Materials with low thermal conductivity have long captured significant attention for heat management applications [1] in many fields including electronics [2], thermoelectrics [3], buildings [4], and aerospace [5]. Since heat can be carried by various excitations [6] inside the material to flow from the hot to the cold side, blocking the transport of primary heat carriers is the key to heat insulation. Due to the requirement of mechanical strength for most application circumstances, solid materials play critical roles in heat insulation, in which electrons and phonons are the major heat carriers. It is then clear that known heat insulators are mostly electronic insulators [7] as well because of the negligible heat transport of electrons.

In a three-dimensional solid, the low-energy acoustic branches, one longitudinal (compressing/stretching lattice) and two shear phonons, provide the main heat carriers [8]. Since most solids have a nearly identical heat capacity of 3 *k_B_*/atom at temperatures above 300 K, known as the Dulong-Petit law, efforts on developing heat insulators are focused on materials either with heavy constituent elements [9] and weak chemical bonds [10] for a low phonon propagating velocity [11] or with bunches of various types of defects [12] and multiscale structures for strong phonon scattering [13–18]. To date, these strategies have led to a successful realization of room-temperature thermal conductivity as low as 0.2 W m^−1^ K^−1^ for dense solids [19].

Elastic properties enable a fundamental understanding of lattice vibrations of solids [20,21]. For example, a usual increase in both strength and density from gas to liquid and then to solid leads to an increase in the velocity of sound waves traveling through these media [22]. Similarly, isotropic solids with a positive Poisson’s ratio are known to usually show a larger elastic modulus than shear modulus. This leads to the relative easiness to shear than to compress/stretch a solid, which further chemically means an easier change in molecular bonding angle than bonding length, which, essentially corresponds to a faster propagation of longitudinal lattice vibrations than that of shear ones.

It is then clearly suggested that populating these easier-shear optical vibrations to produce bond-angle fluctuations fundamentally opens an opportunity for minimizing heat conduction because these shearing vibrations lead to a low optical vibrational frequency for suppressing the frequency of acoustic branches [23,24]. Taking methylene as an example, the planar rocking shows that the two hydrogen atoms undergo rigid-like libration around the central axis defined by the C atom. During such planar rocking, the H-C-H bond angle within the molecule remains nearly unchanged, resulting in a relatively low vibration frequency of 21.6 THz [25] (compared to scissoring vibrations of 43.9 THz, where the hydrogen atoms move in opposite directions). It is defined that the flapping vibrations in crystal systems are those vibration modes analogous to the planar rocking of methylene. Note that the vibrations in crystals are usually more complex than those in molecular examples. For example, the phase differences between vibrational units (such as polyhedra) within lattices must also be considered when discussing the changes in bond angle and length.

According to the theory of deformation, it can be geometrically understood in a two-dimensional space that a deformation gradient can be decomposed into symmetric and antisymmetric parts, and the latter corresponds to a rotation [26]. Once considering the lattice dynamics in a solid at finite temperatures, the trajectory of an optical planar-flapping mode analogously corresponds to the circular shearing deformation fluctuations. This type of vibration might lead to bond-angle fluctuations in chemistry, and might also reduce the frequency of lattice vibrations in physics.

Low bonding strength (i.e., a low force constant) is the core factor underlying low sound velocity, low shear modulus, and low optical mode frequencies. Therefore, to facilitate the effect of flapping vibrations for minimizing heat conduction, the material should then elastically show a low shear modulus. This indicates a strong bond anisotropy (at least locally), which can usually be expected in a low symmetry structure. Surely, a strong bond anisotropy is also helpful for a strong phonon scattering [27].

It is motivated to focus on the low-symmetry orthorhombic Cs_2_ZnI_4_ (space group no. 62, *Pnma*), which has an average atomic mass as heavy as 120 g/mol, as a new heat insulator. It is revealed in single crystals an extremely low thermal conductivity of 0.11 W m^−1^ K^−1^ at room temperature, which is one of the lowest among known heat insulators. This low thermal conductivity stems from the distinct flapping vibrations evident from synchrotron X-ray diffuse scattering, transmission electron microscopy, and Raman spectroscopy characterizations. This work illustrates the importance of flapping vibrations for insulating heat.

## RESULTS AND DISCUSSIONS

**Figure 1.**
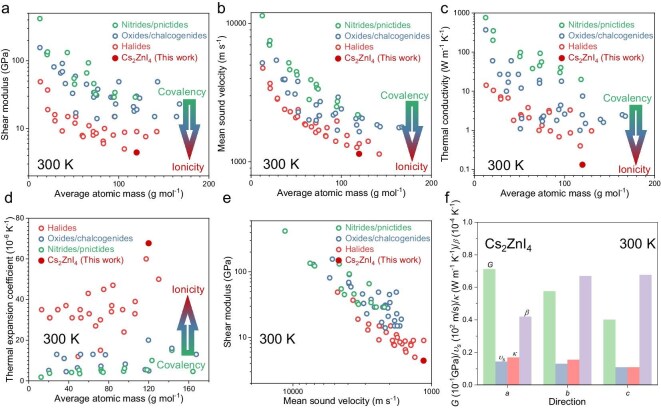
Physical properties. Surveys of average atomic-mass-dependent (a) shear modulus (*G*), (b) mean sound velocity (*v_s_*), (c) thermal conductivity (*κ*), and (d) thermal expansion coefficient (*β*), for nitrides/pnictides, oxides/chalcogenides, halides, and existing heat insulators (partially in the supplementary). (e) Dependency of G on the mean sound velocity and (f) crystallographic-direction-dependent *G, v_s_, κ*, and *β*. The detailed composition and references are given in the supplementary.

Surveys of average atomic mass-dependent shear modulus (*G*), mean sound velocity (*v_s_*), thermal expansion coefficient (*β*), and thermal conductivity (*κ*) for nitrides/pnictides, oxides/chalcogenides, halides, and existing heat insulators are given in Figs 1a–d and S1. Table S1 provides the specific elastic parameters of the single-crystalline Cs_2_ZnI_4_ along the *a*-, *b*-, and *c*-directions. The mechanical properties exhibit significantly anisotropic characteristics in three directions, which correspond to the anisotropy of chemical bonds. At a given average atomic mass, the overall trend of increasing covalency from halides to oxides/chalcogenides and then to nitrides/pnictides drives a corresponding increase in *v_s_* and *κ*, yet induces an inverse response in *β*. Moreover, a low *G* usually leads to a reduced *v_s_* (Fig. 1e), which is even lower in Cs_2_ZnI_4_ than that along the out-of-plane direction in most two-dimensional materials (Fig. S2). All these features are consistent with the common wisdom [11] of weakly-bonded heavy atoms for heat insulators, and this work illustrates in Cs_2_ZnI_4_ that a small *G* is helpful for a low *v_s_* and *κ* but a high *β* (Fig. 1f).

**Figure 2.**
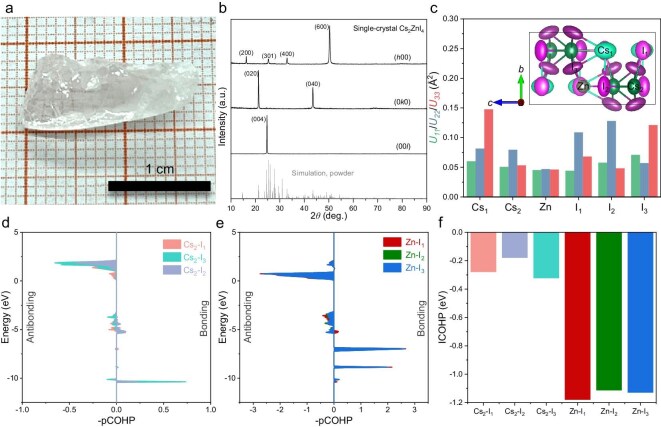
Crystal structure and lattice vibrations. (a) A typical photograph of Cs_2_ZnI_4_ single crystals. (b) X-ray diffraction patterns of the surfaces of crystals with [100], [010], and [001] orientations. (c) Diagonal mean-square displacements of Cs_2_ZnI_4_. Calculated negative projected crystal orbital Hamilton population (–pCOHP) for Cs-I bonds (d), Zn-I bonds (e), and the corresponding integrated COHP (ICOHP) (f).

The experimental details on sample synthesis, characterizations, and property measurements, as well as Density Functional Theory and *ab initio* molecular dynamics calculations, are provided in the Supplementary data. The unique properties of the Cs_2_ZnI_4_ stem primarily from its crystal structure. Figure 2a shows a typical photograph of the single crystal, which enables pellet samples to respectively show [100], [010], and [001] orientations (Fig. 2b). The (301) peak in the (*h*00) X-ray diffraction (XRD) pattern likely arises either from surface scratches introduced during polishing, as the (301) plane has the highest theoretical intensity in random powder samples (Table S2), or from slight misalignment due to its small angular deviation from (*h*00), making it detectable while other intense peaks at larger angles are absent (Table S3). Single-crystal XRD analysis confirms its orthorhombic structure with a *Pnma* space group (Fig. S3 and Table S4), showing three crystallographic sites for iodine (labeled as I_1_, I_2_, and I_3_) and two for cesium (Cs_1_ and Cs_2_) with anisotropic vibrations.

The origin of flapping vibrations in Cs_2_ZnI_4_ mainly arises from two aspects: for a *Pnma* space group, the I_3_ and I_2_ atoms occupy the 8d and 4c sites, respectively. Through inversion and screw symmetry operations, eight equivalent I_3_ atoms and four equivalent I_2_ atoms can be formed. Like the hydrogen atoms in methylene, one pair of the adjacent equivalent iodine atoms form a group, with a certain axis (cesium atoms and zinc atoms) serving as the rotation center. This is the static structural origin for flapping vibrations in real space. On the other hand, the anisotropy of chemical bonds leads to a pronounced directional preference to thermal vibrations, wherein thermal vibrations along directions with weak chemical bonds are large, while those along directions with strong chemical bonds are small. This ultimately leads to significant anisotropies in the thermal vibration ellipsoids of I_3_ and I_2_, providing the lattice dynamical origin for flapping vibrations. Notably, the atomic displacement parameters (ADP, *U_11_, U_22_*, and *U_33_*) demonstrate the anisotropy of lattice vibrations, as visualized in the thermal ellipsoid plot (Fig. 2c). The room-temperature thermal vibrational ellipsoid for each atom is detailed in Fig. S4, with its chemical bonding environments centered in both cations and anions.

The negative projected Crystal Orbital Hamilton Population (–pCOHP) for the Cs-I bonds (Fig. 2d) and Zn-I bonds (Fig. 2e) clearly reveal the bonding and antibonding contributions for various atomic pairs. Notably, some bonds exhibit a distinct antibonding state in the vicinity of the Fermi level. Occupation of these antibonding states increases the total energy of the system, which weakens the chemical bond and enhances the lattice anharmonicity. Furthermore, the Zn-I bond has an integrated COHP (ICOHP) of −1.1 eV, significantly more negative than those of other pairs (Fig. 2f), confirming it as the strongest covalent chemical bond in Cs_2_ZnI_4_. This leads to nearly isotropic atomic displacements for Zn atoms. On the other hand, the Cs-I bond yields an ICOHP of −0.4 eV, indicating a relatively weak chemical bond strength.

To further visualize the chemical bonding nature in Cs_2_ZnI_4_, we calculate the electron localization function (ELF) which can describe the bond type between atoms, as shown in Fig. S5. The large ELF values (>0.5) correspond to covalent bonds and lone pair or inner shell electrons while the smaller ELF values (<0.5) correspond to ionic or metallic bonds. The weak electron localization between neighboring Cs-I suggests ionic interaction, while the relatively large electron localization between neighboring Zn-I implies a more covalent component of chemical bonds.

The temperature-dependent powder XRD (Fig. S6a and b) reveals a positive thermal expansion behavior. Combined analysis of the temperature-dependent XRD (Fig. S6a) and differential scanning calorimetry (Fig. S6c) data reliably demonstrates the material’s thermal stability, with no detectable phase transitions or decomposition occurring below 500 K. The scanning electron microscopy observations equipped with an energy dispersive X-ray spectrometer attest to the good quality of the crystal (Fig. S6d).

**Figure 3.**
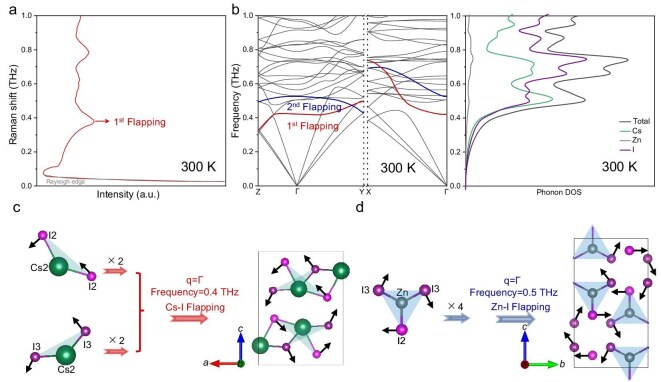
Characteristic flapping frequencies. Raman spectrum (a), and calculated phonon dispersion and atom-resolved phonon density of states (b) for Cs_2_ZnI_4_ at 300 K. Flapping vibrations in Cs_2_ZnI_4_ for cesium atoms (c) and zinc atoms (d) serving as the rotation center.

Phonon spectrum calculations reveal that the planar-like flapping vibrational modes along both the *b* and *c* axes exhibit remarkably low-frequency characteristics [28,29]. Notably, the theoretical frequency of this vibrational mode shows an agreement with the characteristic peak observed at 0.4 THz in room-temperature Raman spectroscopy (Fig. 3a). According to the theoretical analysis, A_1g_, B_1g_, B_2g_, and B_3g_ are the possible Raman active mode symmetries (space group *Pnma*, point group D_2h_). The nice agreement (Fig. S7) in the highest possible phonon frequency between Raman measurements and the first-principles calculations further ensures the measurement accuracy.

In the calculated phonon spectrum with density of state at 300 K (Fig. 3b), two optical branches are marked in red and blue, respectively. At the Γ point, the former corresponds to the flapping vibrations of two I_2_-Cs-I_2_ and two I_3_-Cs-I_3_ units within the *ac*-plane (Fig. 3c), while the latter corresponds to the flapping vibrations of four I_2_-Zn-I_3_ units within the *bc*-plane (Fig. 3d). According to the COHP calculation data (Fig. 2d–f), since the Cs-I bonds are weaker than the Zn-I bonds, the frequency of the Cs-I flapping mode is lower than that of the Zn-I flapping mode.

Since there are neither coupling interactions nor mode hybridization between acoustic and optical phonons along the Γ-Y and Γ-Z directions, their vibrational modes remain mutually independent, and thus the system does not exhibit the avoided crossing phenomenon. The flapping vibrations induced Raman peak at a characteristic frequency as low as 0.4 THz is observed, possibly with a few other low-intensity peaks of higher frequencies within 1 THz. These measurement results nicely agree with the first-principles calculations on the phonon dispersion disclosing extremely low-frequency optical flapping modes (marked in red and blue in Fig. 3b). The rapid increase in density-of-states (DOS) around 0.4 THz originates from the contribution of flapping vibrations. These modes largely contribute to the phonon DOS due to the intensive vibrations of Cs and I at around 0.5 THz.

**Figure 4.**
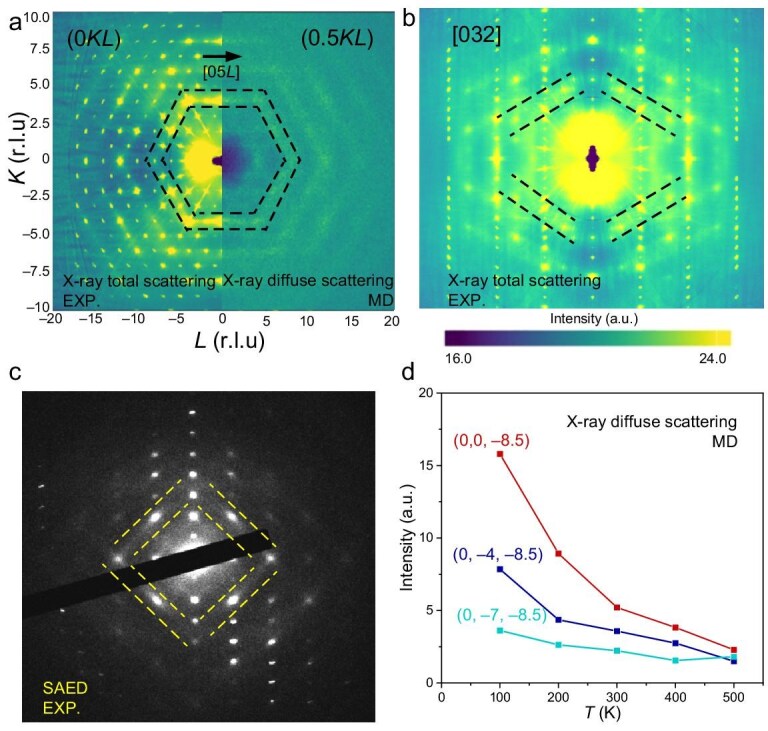
(a) Room-temperature three-dimensional volumes of X-ray diffuse scattering for single-crystal Cs_2_ZnI_4_ at *Q* = (0*KL*), along with the simulation results from molecular dynamics (MD) at *Q* = (0.5*KL*). (b) Experimental three-dimensional X-ray diffuse scattering slice along the [032] direction. (c) Selected area electron diffraction (SAED) pattern. (d) Temperature-dependent X-ray diffuse signal at *Q* = (0, 0, –8.5), (0, –4, –8.5), and (0, –7, –8.5) based on MD simulations.

These flapping vibration-induced displacements of atoms are observed by the three-dimensional single crystal synchrotron X-ray diffuse scattering measurements. The reciprocal-space diffuse scattering data were collected at the QM2 beamline at the Cornell High Energy Synchrotron Source. More details of data processing are provided in supplementary data [30]. In X-ray single-crystal total scattering experiments, each bright spot observed in reciprocal space (Fig. 4a) is a Bragg diffraction point, reflecting the long-range order of equilibrium positions of atoms. When those atoms deviate from their long-range periodic positions (e.g. due to lattice defects or optical phonons), it gives rise to so-called diffuse scattering signals across the reciprocal space (e.g. diffuse rods). If the atoms (or atomic clusters) deviating from their equilibrium positions form one-, two-, or three-dimensional short-range order in real space, the diffuse scattering signals will correspondingly exhibit two-, one-, or zero-dimensional geometric characteristics in reciprocal space.

In our experiments, the diffuse scattering signals (left panel in Fig. 4a, marked by black dashed lines) reveal the characteristic diffuse rods along certain directions. For example, the [05*L*] direction in the reciprocal space is related to the large *U*_22_ of I_2_ atoms in real space due to the flapping vibrations. The experimental observations can be well reproduced by the large-scale molecular dynamics (MD) simulation (right panel in Fig. 4a), which confirms the intrinsic nature of thermal diffuse scattering. Note that the Bragg diffraction peaks are suppressed when *H* equals half-integer values (e.g. 0.5), at which the diffuse scattering signals become more pronounced.

Similarly, diffuse signals can also be observed along the [032] direction (Fig. 4b, marked by black dashed lines). We further employed selected-area electron diffraction (SAED) to cross-validate the observed diffuse scattering signals. The streak-like contrast between SAED spots indicates the presence of diffuse scattering information (Fig. 4c, marked by the yellow dashed lines), whose geometric features are consistent with the X-ray scattering experimental results. It is worth noting that the sample is relatively sensitive to electron beam irradiation. Although the diffraction data were acquired using as weak an electron beam and as short an exposure time as possible, some subtle changes in the sample may still have occurred during acquisition (specifically manifested as an incomplete correspondence of extinction spots between the XRD and electron diffraction data). Nevertheless, both experiments and simulations indicate that the diffuse scattering information is indeed consistently present.

Given that the MD results well reproduce the experimental data at 300 K, we have calculated diffuse scattering data at different temperatures using MD simulations (Fig. 4d) to discuss the relation between diffuse scattering, dynamical phenomenon, and the flapping. We argue that these diffuse signals are dynamic and phonon-related, rather than static defect structures. Figure 4d displays the (*HKL*)-dependent diffuse scattering intensity at different temperatures. It can be found that the diffuse scattering arising from short-range order exhibits a clear temperature dependence, indicating that it is primarily phonon-dominated.

The calculated mode Gruneisen parameters at 300 K (Fig. S8a) are used to estimate the anharmonicity parameter. Certain phonon modes of ∼0.5 THz exhibit significantly negative mode Gruneisen parameters, which are attributed to the rotation motion of the flapping vibrations. In general, the frequency dependence of the phonon scattering rate approximately follows *ω*², indicating that three-phonon scattering is the dominant scattering mechanism. It is worth noting that the phonon scattering rates are enhanced near the frequencies of the first and second flapping modes (Fig. S8b), due to the enhanced scattering phase space.

The measured heat capacity at constant pressure (*C*_p_) shows an agreement with that predicted according to the Dulong-Petit law at room temperature and above (Fig. S9a). Therefore, to maintain consistency in the thermal conductivity estimation, the use of the Dulong-Petit limit in this work is reasonable and conservative in the temperature range of 200–425 K. The existence of extremely low-frequency flapping vibrations is therefore expected to largely contribute to *C*_p_ beyond that predicted by the Debye model, which is observed at temperatures much lower than the Debye temperature (*Θ*_D_) of 94 K in Fig. S9b. Taking into account the significant increase in phonon DOS around the frequency of flapping modes, the increase in *C*_p_ can then be well reproduced, indicating quite similar lattice dynamics for the low-temperature phase [31].

The calculated frequency-dependent group velocity (Fig. S10), particularly for longitudinal acoustic branches along Γ-A direction, reveals a significant reduction once its frequency approaches the characteristic one of flapping modes. As these flapping vibrations are deeply wedged into a low-frequency range, a significant resistance to heat conduction can be expected because of the large reduction in group velocity of acoustic phonons (the primary heat carriers).

**Figure 5.**
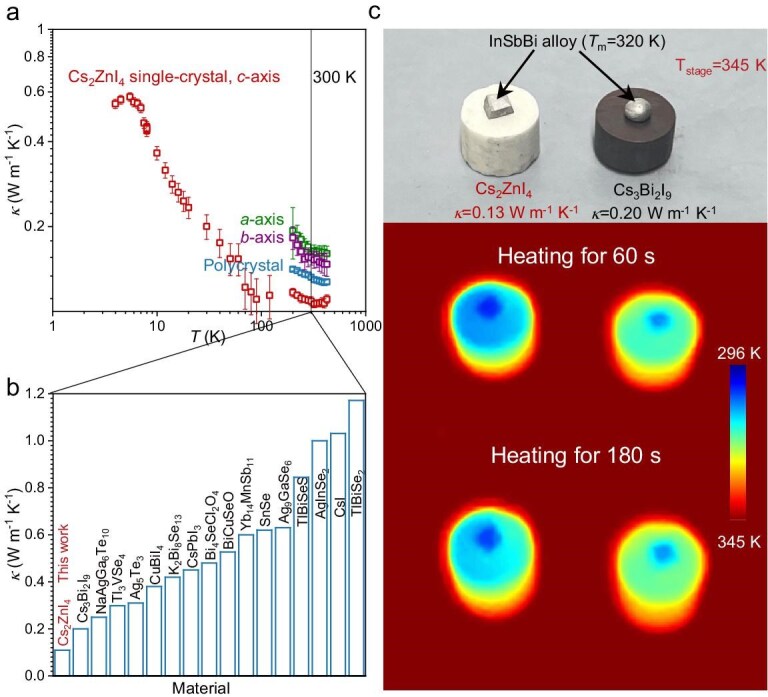
Heat insulation performance. (a) Temperature-dependent thermal conductivity for Cs_2_ZnI_4_, and (b) a comparison to that of existing dense heat insulators [14,28,29,34–39]. (c) A comparison in heat insulation between Cs_2_ZnI_4_ and Cs_3_Bi_2_I_9_ [19] with the same-size cylinders placed on a heating stage monitored by optical and infrared cameras for different heating times.

Furthermore, due to the strongly suppressed sound velocity, single-crystal Cs_2_ZnI_4_ along the *c*-axis exhibits an extraordinarily low *κ* of 0.11 W m^−1^ K^−1^ at 300 K (Fig. 5a and b, and the measurement setup and the raw data are given in Figs S11–S13). This value is extremely close to the theoretical minimum described by the Cahill model, indicating that its heat transport has approached the minimum limit. The measured thermal conductivity obtained by the guarded hot-plate method agrees with that from laser flash analysis within a relative deviation of less than 5% (Fig. S14 and Tables S5 and S6), confirming the reliability of the reported thermal conductivity data. It can be seen from Table S1 that the anisotropy in thermal conductivity is consistent with that of sound velocity, both exhibiting a decreasing trend along the crystallographic axes from *a* to *b* then to *c*. This indicates that thermal conductivity is predominantly dominated by the long-wavelength acoustic phonons. The sound velocity in the *a*-direction is ∼1.3 times that in the *c*-direction. According to the Slack model, thermal conductivity is proportional to the cube of the sound velocity. However, the thermal conductivity in the *a*-direction is less than 1.3^3^ times that in the *c*-direction. This suggests that the rapid decrease in phonon group velocity along the *a*-direction still contributes to the reduction of thermal conductivity in that direction.

The calculation of thermal conductivity indicates that the thermal conductivity of Cs_2_ZnI_4_ is predominantly dominated by particle-like phonon transport at room temperature, while the contribution from wave-like phonon transport is not significant (Table S7). This explains the experimentally observed decrease in thermal conductivity with increasing temperature below room temperature. It is worth noting that the calculated anisotropy of thermal conductivity is relatively weak, which may be attributed to the possible inaccuracy in phonon renormalization. As shown in Fig. S15, the anisotropy of sound velocity after phonon renormalization is also not as pronounced as the experimentally measured values. We recognize that these discrepancies likely arise from the limitations of the second-order effective harmonic model in fully capturing the higher-order anharmonic effects of certain phonon modes, particularly for systems with large mean momentum densities at elevated temperatures.

The thermal conductivity measurements at low temperatures exhibit a distinct crystal peak, indicating that the sample possesses high crystallinity. Due to the rapid increase in heat capacity at low temperatures, the thermal conductivity below 5.5 K rises with increasing temperature and reaches a peak at 5.5 K, with the magnitude of the peak determined by the grain size of the measured sample. When the temperature exceeds 5.5 K, the thermal conductivity decreases with increasing temperature, indicating that phonon scattering is dominated by three-phonon scattering processes. The strong ionicity and ionic insulation behavior (2 × 10^−10^ S/cm) as well as the large optical band gap (4.3 eV) shown in Fig. S16, ensure the absence of electrons contributing to heat conduction in Cs_2_ZnI_4_.

The heat insulation performance of Cs_2_ZnI_4_ is further compared to that of known Cs_3_Bi_2_I_9_ [19] (synthesized as well in this work) with a room-temperature *κ* of 0.20 W m^−1^ K^−1^ (Fig. 5c) and Bi_4_SeCl_2_O_4_ [27,32,33] (Fig. S17). The XRD data for the cylindrical samples are shown in Fig. S18. Both cylinders of the same size with cubes of an InSbBi alloy (melting point *T*_m_ = 320 K) on the top are placed on a heating stage of 345 K, with temperatures monitored by an infrared camera. Compared with the Cs_3_Bi_2_I_9_ cylinder, the upper surface temperature of the Cs_2_ZnI_4_ cylinder is much lower, and the InSbBi alloy placed on top remains unmeted, indicating that this material exhibits better thermal insulation performance. Note that Cs_3_Bi_2_I_9_ exhibits higher emissivity compared to Cs_2_ZnI_4_, which could lead to underestimation of actual temperature measurements. Nevertheless, experimental observations revealed that the alloy deposited on the top of the Cs_3_Bi_2_I_9_ cylinder melted, whereas the alloy on the Cs_2_ZnI_4_ cylinder remained in a solid phase. These comparative experimental results demonstrate that temperature measurement deviations caused by emissivity differences are not the critical factor influencing the observed melting phenomenon.

## CONCLUSION

Inspired by the common wisdom that shearing is usually easier than stretching/compressing, this work chemically constructs building blocks of flapping vibrations and physically utilizes the low characteristic frequency feature for eventually blocking phonon transport. It is illustrated that the low-symmetry Cs_2_ZnI_4_ crystal of heavy elements enables flapping vibrations that lead to very low shear moduli, sound velocities, and thermal conductivity. We hope the strategy here could broaden the pathway for advancing thermally functional materials for both heat preservation and energy conversion (such as thermoelectrics) applications.

## METHODS

Detailed methods can be found in the online supplementary information.

## Supplementary Material

nwag256_Supplemental_File
