# Supplementary material for: Flapping vibrations contributed to heat insulation in Cs2ZnI4
Source: Natl Sci Rev. 2026 May 1;13(12):nwag256. doi: 10.1093/nsr/nwag256 (PMC13309929; doi:10.1093/nsr/nwag256)
Supplement: nwag256_Supplemental_File [file nwag256_supplemental_file.pdf]

Supplementary for "Flapping vibrations contributed to heat insulation in Cs_2ZnI_4"

Linjie Wu[1,†], Pengfei Nan[2,†], Long Yang[1,†], Zhiwei Chen[1,*], Changyuan Li[1], Qingyu Bai[1], Hongzheng Wang[2], Sui Ting Tai[3], Chen Wang[3], Binghui Ge[2], Yue Chen[3], Jun Luo[1,*] and Yanzhong Pei[1,*]

[1]Interdisciplinary Materials Research Center, School of Materials Science and Engineering, Tongji University, Shanghai, 201804, China.

[2]Institutes of Physical Science and Information Technology, Anhui University, Hefei, 230601, China.

[3]Department of Mechanical Engineering, The University of Hong Kong, Hong Kong, China.

***Corresponding authors.** E-mails: 14czw@tongji.edu.cn; junluo@tongji.edu.cn; yanzhong@tongji.edu.cn

[†]Equally contributed to this work.

Section S1: Physical properties

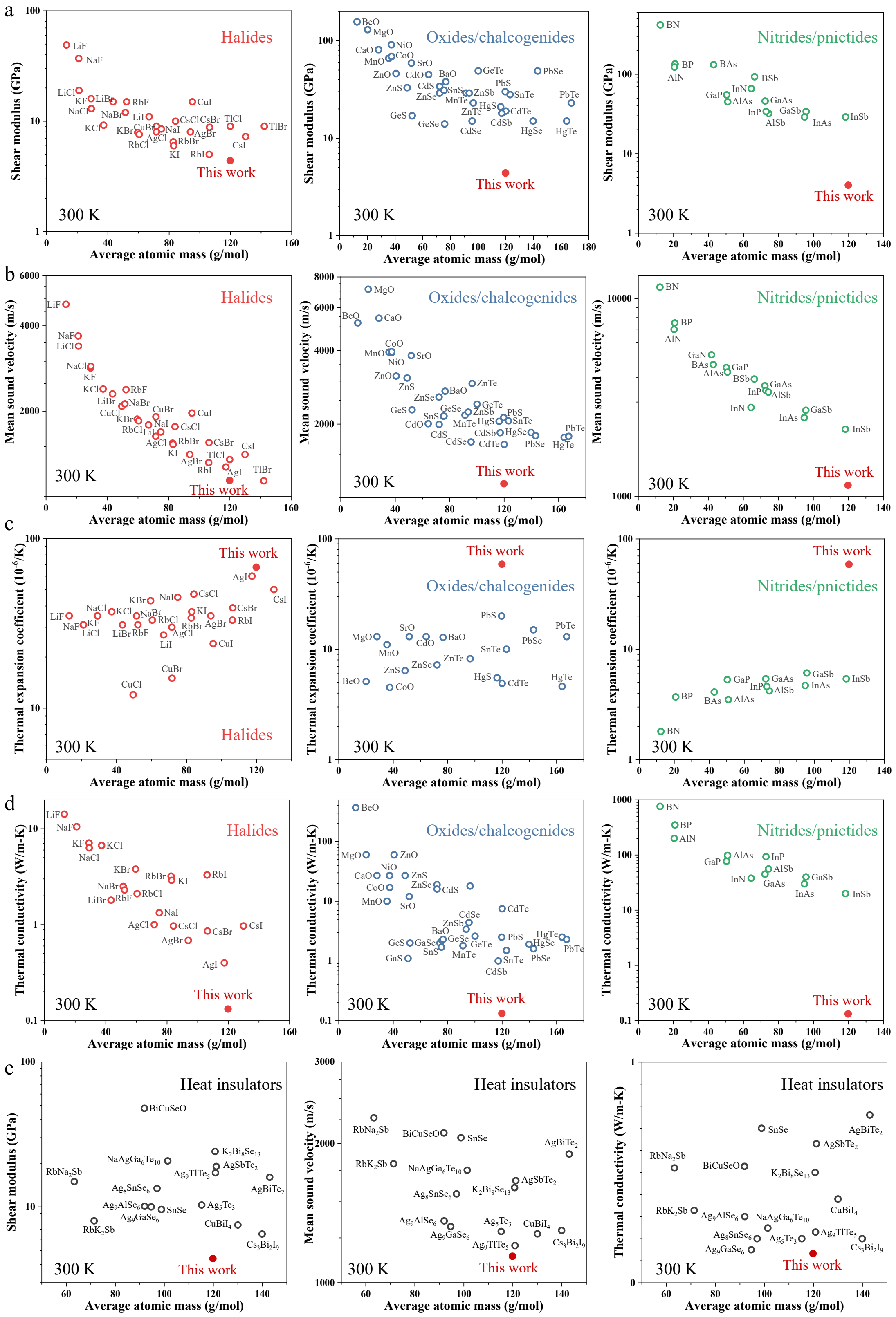

Figure S1. Surveys of average atomic mass dependent (a) shear modulus (G), (b) mean sound velocity (v_s), (c) thermal expansion coefficient (β), (d) thermal conductivity (κ) for nitrides/pnictides, oxides/chalcogenides, halides[1-128] and (e) comparison with existing heat insulators[114,129-140].

The additional physical parameters listed in **Table S1** were estimated by the following equations (S1) to (S6). The shear modulus (G), bulk modulus (B) and Young's modulus (E) are determined through the formula,

$$G = \rho v_T^2 \tag{S1}$$

$$B = \rho v_L^2 - \frac{4}{3}G \tag{S2}$$

$$E = 2G(1+r) \tag{S3}$$

where ρ is the mass density, v_T is the transverse sound velocity and v_L is the longitudinal sound velocity. The Poisson's ratio (r) and Gruneisen parameter (γ) are estimated by

$$r = \frac{\left(\frac{v_T}{v_L}\right)^2 - 2}{2\left[\left(\frac{v_T}{v_L}\right)^2 - 1\right]} \tag{S4}$$

$$\gamma = \frac{3}{2}\left(\frac{3v_L^2 - 4v_T^2}{v_L^2 + 2v_T^2}\right) \tag{S5}$$

The Debye temperature (θ_D) is evaluated as follows,

$$\theta_D = \frac{\hbar}{k_B}\left(\frac{6\pi^2}{V}\right)^{\frac{1}{3}}\left(\frac{2}{3v_T^3} + \frac{1}{3v_L^3}\right)\Big)^{-\frac{1}{3}} \tag{S6}$$

where $\hbar$ is the reduced Planck constant, k_B is the Boltzmann constant and V is the average atomic volume. The above equations are included in the revised supporting information.

Table S1. The physical parameters (Transverse sound velocity v_t, Longitudinal sound velocity v_l, Mean sound velocity v_s, Shear modulus G, Bulk modulus B, Young's modulus E, Poisson ratio r and Gruneisen constant γ) and thermal conductivity κ at room temperature for Cs_2ZnI_4.

Directions	v_t (m/s)	v_l (m/s)	v_s (m/s)	G (GPa)	B (GPa)	E (GPa)	r	γ	κ (W/m-K)
a	1290	2314	1437	7.1	13.4	18.2	0.27	1.6	0.17
b	1161	2411	1305	5.8	17.2	15.6	0.35	2.1	0.16
c	968	1936	1086	4.0	10.7	10.7	0.33	2.0	0.11

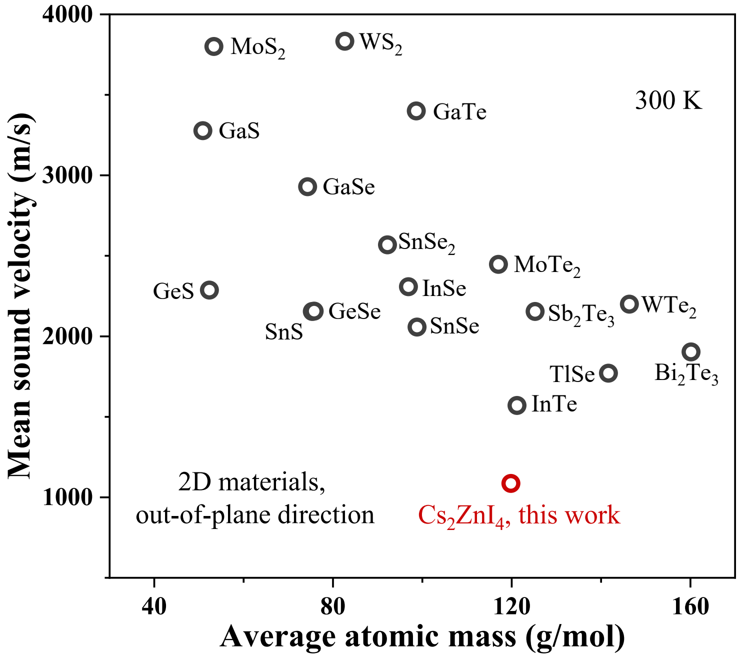

Figure S2. Room-temperature mean sound velocity of Cs_2ZnI_4, as compared to that of two-dimensional materials along the out-of-plane directions[71,75,141-153].

Section S2: Sample synthesis and phase purity

High-purity CsI (99.9%, Aladdin) and ZnI_2 (99.99%, Aladdin) were mixed in a stoichiometric ratio, loaded in a quartz ampule, and sealed under high vacuum. To synthesize polycrystalline Cs_2ZnI_4, the sealed ampule was heated to 923 K, held at this temperature for 5 hours, and subsequently quenched in cold water, followed by annealing at 523 K for 48 hours. The resulting ingot was hand-ground into fine powder and then hot-pressed at 503 K for 60 minutes under a uniaxial pressure of approximately 50 MPa. Dense pellets with a relative density exceeding 97% of the theoretical value were obtained and subsequently fabricated into various

geometries for different characterization measurements.

A vertical gradient freeze method was used for the growth of single-crystalline Cs_2ZnI_4. The ampoule was placed in a vertical temperature gradient furnace and the temperature (T_0) located at the tip of the ampoule was heated to 923 K and held at this temperature for 5 hours. From 833 K to 673 K, the ampoule was slowly cooled down at a rate of ~ 0.5 K/h, and then slowly cooled to room temperature.

The $Cs_3Bi_2I_9$ sample was synthesized by mixing high-purity CsI (99.9%, Aladdin) and BiI_3 (99.99%, Aladdin) in stoichiometric proportions. The mixture was loaded into quartz ampoules and sealed under vacuum. The ampoules containing the powder mixture were heated to 973 K and held for 6 hours, then quenched in cold water. Subsequently, the ingot, which remained sealed in a vacuum, was annealed at 573 K for 48 hours. The annealed ingot was then hand-ground into fine powders and hot-pressed at 550 K for 20 mins. As a result, a dense bulk sample with a diameter of about 12 mm, a thickness of about 8 mm, and a density greater than 97% of the theoretical density was obtained.

The $Bi_4SeCl_2O_4$ sample was synthesized by mixing high-purity $BiCl_3$, Bi_2O_3, and Bi_2Se_3 powders in stoichiometric proportions. This mixture was then compacted into a bulk under a uniaxial pressure of 80 MPa at room temperature for 10 minutes. The bulk was subsequently vacuum-sealed in a quartz ampoule and subjected to a solid-state reaction at 1073 K for 48 hours, followed by slow cooling in the furnace to room temperature. The resulting pellets were then hand-ground into fine powders and hot-pressed at 923 K under 90 MPa for 60 mins. Consequently, a dense bulk sample with a diameter of about 12 mm, a thickness of about 8 mm, and a density greater than 95% of the theoretical density was obtained.

Temperature-dependent X-ray diffraction (XRD, Rigaku SmartLab, Cu-Kα radiation) of Cs_2ZnI_4 powders shows no phase transition or phase decomposition up to 500 K. The orientations of single-crystalline Cs_2ZnI_4 were characterized by XRD (DX2000, Cu-Kα radiation). The composition of single crystalline Cs_2ZnI_4 was characterized by Scanning Electronic Microscopy (SEM, Phenom Pro) equipped with an Energy Dispersive X-ray Spectrometer (EDS), confirming the homogenous composition. The selected area electron diffraction (SAED) pattern was collected by the JEM-F200 microscope at 200 kV.

Table S2. X-ray diffraction peaks of Cs_2ZnI_4 (ICSD #82932), including Miller indices (*hkl*), 2θ positions, and relative intensities (*I*).

hkl	2θ/ (°)	*Intensity* (%)
200	16.36	1.21
301	25.41	100
400	33.06	0.45
600	50.53	5.67

Table S3. X-ray diffraction peaks of Cs_2ZnI_4 (ICSD #82932), including Miller indices (*hkl*), 2θ positions, relative intensities (*I*) and the angle between (*hkl*) plane and the (*h*00) plane.

hkl	2θ/ (°)	*Intensity* (%)	Angle (°)
301	100	100	14.006
122	99.82	99.82	71.590
004	90.93	90.93	90
221	85.23	85.23	53.576
303	59.69	59.69	36.808

Section S3: Single-crystal structure analysis

The diffraction data of single crystals were collected by a single crystal X-ray diffractometer (Bruker D8 VENTURE diffractometer with a PHOTON II CCD detector, Mo-Kα radiation). The crystal was maintained at 300 K during data collection. The crystal structure was subsequently solved and refined using SHELXT and OLEX2[154,155](**Figure S3**). Detailed crystallographic parameters are provided in **Table S3**.

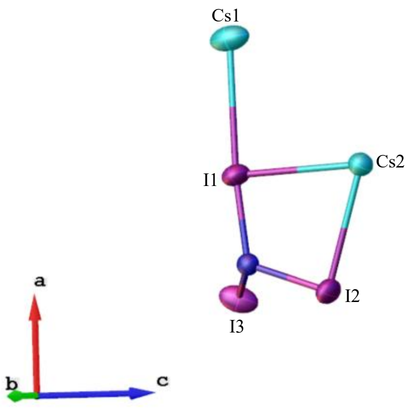

Figure S3. Thermal-vibrational ellipsoids for each atom in Cs_2ZnI_4 at 300 K, as solved using SHELXT.

Table S4. Crystallographic information according to single-crystal X-ray diffraction refinements at 300 K.

Formula	Cs_2ZnI_4
Molar mass, g/mol	838.79
Crystal size, mm^3	0.02 × 0.19 × 0.27

Crystal system	orthorhombic
Space group	*Pnma*
T, K	300
a, Å	10.8227
b, Å	8.3074
c, Å	14.4721
α, °	90
β, °	90
γ, °	90
V, Å^3	1301.2
Z	4
D_{calcd}, kg·m^{-3}	4.282
F (000)	1408
μ, mm^{-1}	16.828
θ range, °	2.81-28.30
reflections collected	27568
independent reflections (R_{int})	1718 (0.0455)
reflections observed [$I > 2\sigma(I)$]	1595
data/restraints/parameters	1718/0/41
R_1, wR_2 ($I > 2\sigma(I)$)	0.0361, 0.0925
R_1, wR_2 (all data)	0.0383, 0.0940
GooF on F^2	1.071
$\Delta\rho_{max}$, $\Delta\rho_{min}$, e·Å^{-3}	1.438/-1.353
position $x/y/z$ of Cs_1	0.13200/0.250000/0.60416
position $x/y/z$ of Cs_1	0.36800/0.750000/0.10416
position $x/y/z$ of Cs_1	0.63200/0.250000/0.89584
position $x/y/z$ of Cs_1	0.86800/0.750000/0.39584
position $x/y/z$ of Cs_2	0.02366/0.750000/0.83182
position $x/y/z$ of Cs_2	0.47634/0.250000/0.33182
position $x/y/z$ of Cs_2	0.52366/0.750000/0.66818
position $x/y/z$ of Cs_2	0.97634/0.250000/0.16818
position $x/y/z$ of Zn	0.23524/0.250000/0.92262
position $x/y/z$ of Zn	0.26476/0.750000/0.42262
position $x/y/z$ of Zn	0.73524/0.250000/0.57738
position $x/y/z$ of Zn	0.76476/0.750000/0.07738
position $x/y/z$ of I_1	0.00326/0.750000/0.09816
position $x/y/z$ of I_1	0.49674/0.250000/0.59816
position $x/y/z$ of I_1	0.50326/0.750000/0.40184
position $x/y/z$ of I_1	0.99674/0.250000/0.90184
position $x/y/z$ of I_2	0.18773/0.750000/0.59251
position $x/y/z$ of I_2	0.31227/0.250000/0.09251
position $x/y/z$ of I_2	0.68773/0.750000/0.90749
position $x/y/z$ of I_2	0.81227/0.250000/0.40749
position $x/y/z$ of I_3	0.17294/0.50449/0.65512
position $x/y/z$ of I_3	0.82706/0.50449/0.84488
position $x/y/z$ of I_3	0.32706/0.49551/0.34488
position $x/y/z$ of I_3	0.67294/0.49551/0.15512
position $x/y/z$ of I_3	0.82706/0.99551/0.65512
position $x/y/z$ of I_3	0.17294/0.00449/0.34488
position $x/y/z$ of I_3	0.67294/0.00449/0.15512
position $x/y/z$ of I_3	0.32706/0.99551/0.84488
mean-square displacements $U_{11}/U_{22}/U_{33}/U_{12}/U_{13}/U_{23}$ of Cs_1 (Å^2)	0.0601/0.0815/0.1477/0/0.0183/0
mean-square displacements $U_{11}/U_{22}/U_{33}/U_{12}/U_{13}/U_{23}$ of Cs_2 (Å^2)	0.0507/0.0796/0.0532/0/-0.0023/0
mean-square displacements $U_{11}/U_{22}/U_{33}/U_{12}/U_{13}/U_{23}$ of Zn (Å^2)	0.0454/0.0470/0.0461/0/0.0006/0
mean-square displacements $U_{11}/U_{22}/U_{33}/U_{12}/U_{13}/U_{23}$ of I_1 (Å^2)	0.0441/0.1089/0.0680/0/0.0100/0
mean-square displacements $U_{11}/U_{22}/U_{33}/U_{12}/U_{13}/U_{23}$ of I_2 (Å^2)	0.0577/0.1281/0.0482/0/0.0108/0
mean-square displacements $U_{11}/U_{22}/U_{33}/U_{12}/U_{13}/U_{23}$ of I_3 (Å^2)	0.0709/0.0571/0.1211/-0.0340/-0.0243/0.0062

The three-dimensional synchrotron X-ray diffuse scattering measurements were carried out at the QM2 beamline at the Cornell High Energy Synchrotron Source (CHESS). The Cs_2ZnI_4 single crystal was measured at 300 K using an incident beam energy of 50.0 keV. A PILATUS 6M Silicon two-dimensional detector was mounted behind the sample perpendicular to the primary beam path with a sample-to-detector distance of 400.6346 mm. During the measurements, the single crystal was continuously rotated by 365° with a 0.1° step about an axis perpendicular to the beam. The exposure time for each frame was 0.1 s. Three sets of rotation images were collected for the Cs_2ZnI_4 sample with the angle slightly changed of two extra distinct axes to fill in detector gaps. The resulting images were stacked into a three-dimensional array and transformed into reciprocal space coordinates using the software packages NXRefine and CCTW (Crystal Coordinate Transformation Workflow) within NeXpy[156-158]. The reciprocal-space data obtained covered a Q range of approximately ±14 $Å^{-1}$ in all directions.

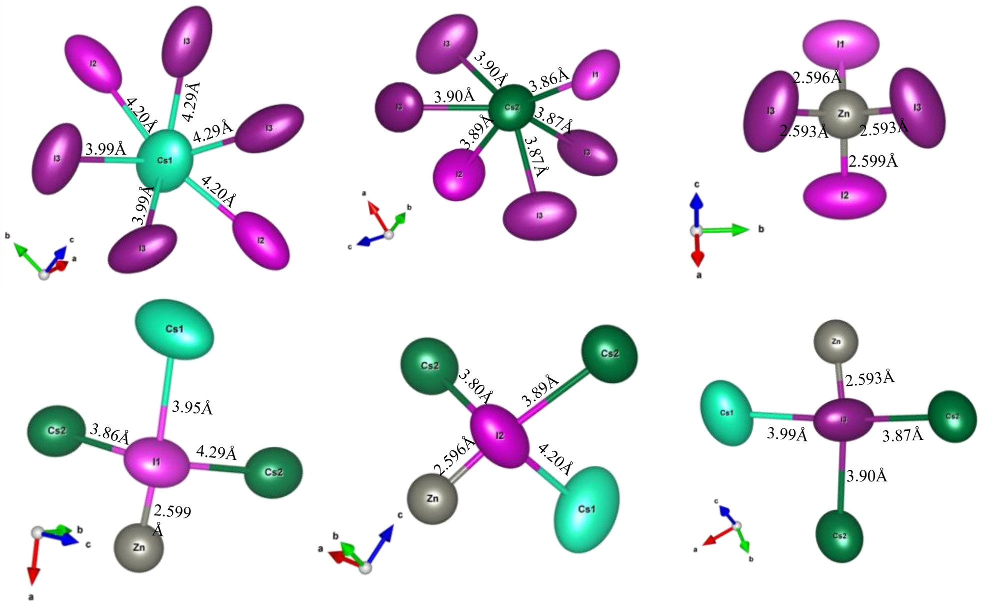

Figure S4. The coordination environment of each cation and anion in Cs_2ZnI_4 at 300 K.

Section S3: Density functional theory (DFT) calculation details

The phonon properties at 300 K were calculated using the Phonopy and Phono3py packages[159] based on 2nd and 3rd order interatomic force constants (IFCs) constructed using the temperature-dependent effective potential (TDEP) approach[160]. The unit cell of Cs_2ZnI_4 was fully relaxed under the density functional theory (DFT) calculation using projector-augmented wave (PAW) pseudopotentials[161] and revised Perdew-Burke-Ernzerhof (PBE) generalized gradient approximation exchange-correlation functional for densely packed solids (GGA-PBEsol)[162] as implemented in the Vienna ab-initio simulation package (VASP)[163]. The structure was fully relaxed until the force and energy reached the convergence criteria of 10^{-2} eV/Å and 10^{-7} eV, respectively. Phonon density of states (DOS), dispersion curves, and group velocities at 300 K were calculated with the Phonopy package using the effective 2nd order IFCs. Forces and energies of the extracted samples were evaluated using DFT and then fitted for IFCs using the Hiphive package[164]. In the relaxation process and for the evaluation of atomic force and energy, we used a kinetic energy cutoff of 600 eV to truncate the plane wave basis set in the DFT calculations. A Γ-centered Monkhorst-Pack scheme with a sampling spacing of about $2\pi\times0.03$ $Å^{-1}$ in the first Brillouin zone was adopted.

Electron localization functions (ELF) were calculated as implemented in the VASP package[165]. Crystal Orbital Hamilton Population (COHP) was using the available software LOBSTER[166] with charge density results from the self-consistent field calculation of the above DFT computation. Gruneisen parameters and phonon scattering rates at 300 K were computed using the 3rd order IFCs with the Phono3py package. Lattice thermal conductivity was computed by solving the direct linearized Boltzmann transport equation as implemented in Phono3py packages, using an $8\times8\times8$ q-points mesh and the 3rd order IFCs obtained above. The coherent contribution to lattice thermal conductivity was calculated with the Unified Theory formula derived from Wigner Transport equation [167] as integrated in the Phono3py packages. The atomic models were visualized using VESTA[168], while the AIMD trajectories were visualized by OVITO[169].

For the analysis of short-range order, molecular dynamics (MD) simulations were performed using a 7×9×5 supercell containing 8820 atoms. The simulations utilized the MACE-Omat potential[170]. The system was first equilibrated in the Nosé-Hoover NVT ensemble for 1.25 ps, followed by a production run in the NVE ensemble for 7.25 ps. The atomic velocities were collected at an interval of 10.0 fs during the final 6.0 ps of the trajectory for subsequent analysis. All MD simulations were carried out using the Large-scale Atomic/Molecular Massively Parallel Simulator (LAMMPS) package[171] with a time step of 1.0 fs. The reciprocal space resolution from MD is $\Delta Q \approx 0.08$ $Å^{-1}$.

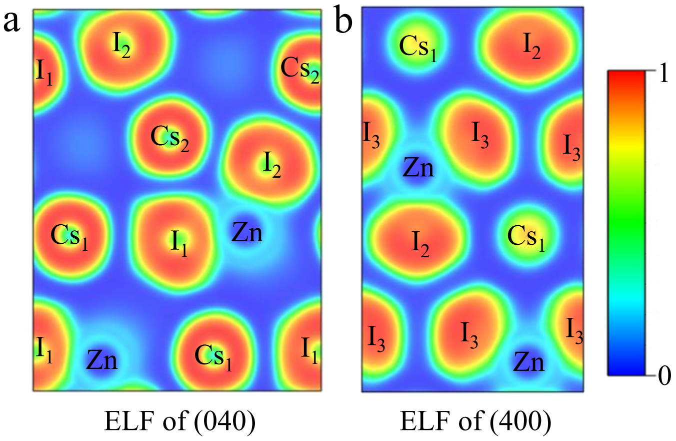

Figure S5. Electron localization function (ELF) of Cs_2ZnI_4 for (040) plane (a) and (400) plane (b) visualized using VESTA.

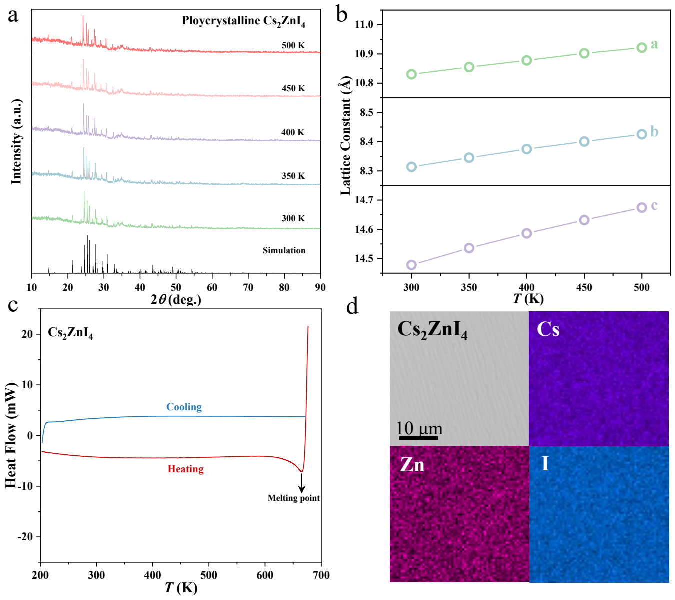

Figure S6. Temperature-dependent powder X-ray diffraction patterns for Cs_2ZnI_4 (a), Temperature-dependent lattice parameters show a positive thermal expansion coefficient along the crystallographic *a*-axis, *b*-axis, and *c*-axis (b), DSC measurement results (c), SEM and EDS images of the cleaved surfaces of Cs_2ZnI_4 (d).

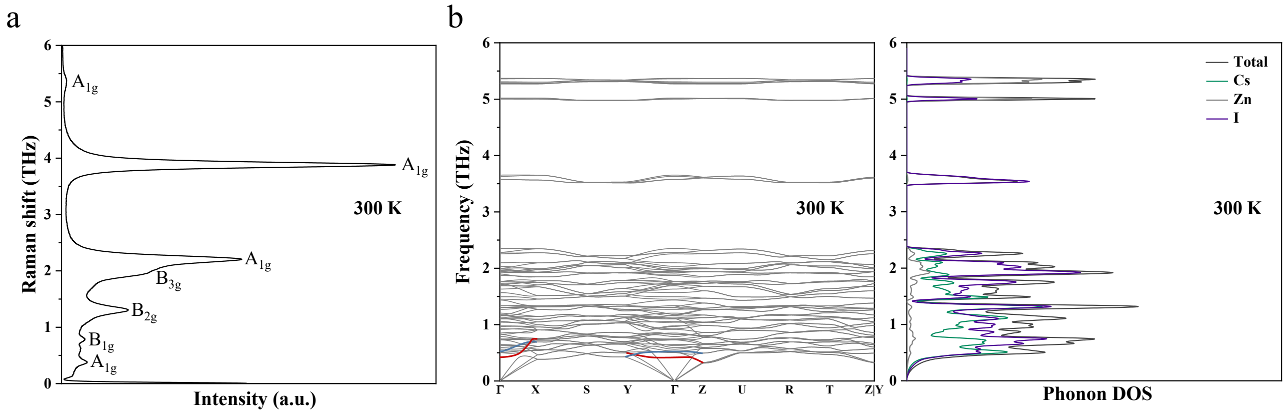

Figure S7. (a) Room-temperature Raman spectra. (b) Calculated phonon spectrum at 300 K with the lowest-frequency optical branch marked in red and atom-resolved phonon density of states in Cs_2ZnI_4.

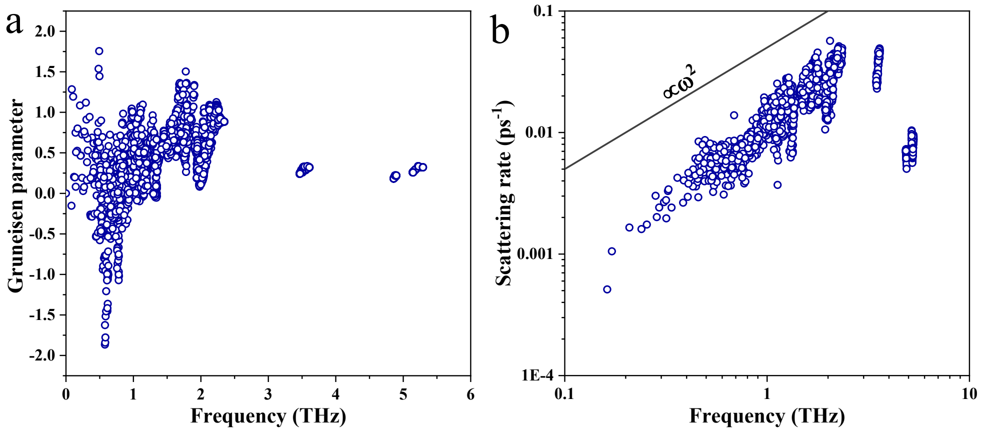

Figure S8. (a) Calculated Gruneisen parameter of Cs_2ZnI_4 at 300 K. (b) Frequency-dependent phonon scattering rate at 300 K.

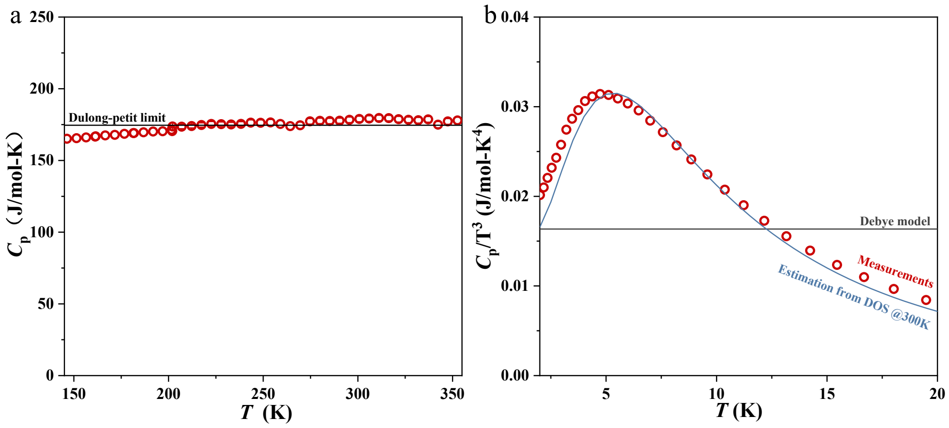

Figure S9. (a) High temperature C_p versus T plot of Cs_2ZnI_4. (b) Temperature-dependent heat capacity normalized by T^3 and estimation from DOS.

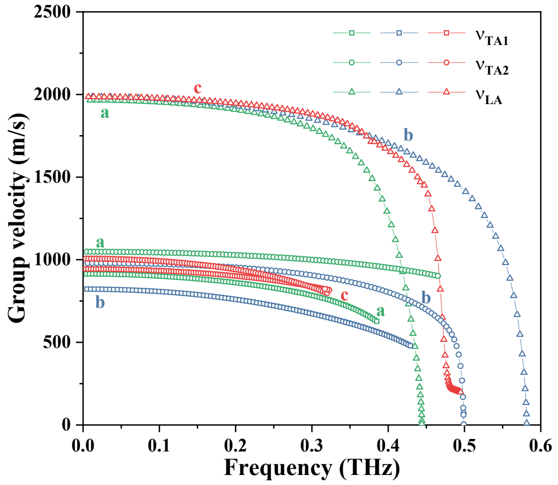

Figure S10. Calculated phonon frequency dependent group velocity.

Section S4: Thermal transport property measurements

In the temperature range from 200 to 425 K, the thermal conductivity (κ) was measured by the laser flash method (Netzsch LFA467) via $\kappa=\rho C_p\lambda$, where ρ is the density, C_p is the heat capacity (using the Dulong-Petit limit of heat capacity in this work), and λ is the thermal diffusivity. All samples were ~ 1 mm thick and tested in an argon atmosphere. From 4 to 150 K, the thermal conductivity was measured by the steady-state method (Multifield Technology Co.) via $\kappa=PL/(\Delta TS)$, where P is the heater power, L is the length between two thermocouple contacts, ΔT is the temperature difference after stabilization of heating and before heating and S is the cross-sectional area of the sample. The sample used in the test is about 2.5×3.5×5.1 mm^3 in size. The heat capacity was measured in a physical properties measurement system (PPMS, Quantum Design) from 2 to 352 K. The steady-state method is primarily used for thermal conductivity measurements below 200 K, and this method is more accurate in the low-temperature region where thermal radiation is not significant (e.g., below 30 K). In this work, a combination of E-type thermocouples and a nanovoltmeter was employed for temperature measurement, ensuring a signal-to-noise ratio greater than 10. **Figure S13** shows the raw temperature curves for thermal conductivity measurements near the crystal peak temperature. It can be observed that after applying the heating power, the temperature curves reached thermal equilibrium, ensuring an accurate estimation of the maximum

temperature difference. The total uncertainty originates from: thermal diffusivity measurement (due to signal fitting and laser energy fluctuations), specific heat (PPMS calibration error), sample thickness and density (±0.5%).

Thermal conductivity measurements were performed using a DRPL-3 high-precision thermal conductivity meter (Xiangtan Xiangyi Instrument Co., Ltd.), based on the guarded hot-plate method and the principle of steady-state planar heat conduction. During the measurement, a constant temperature was applied to one side of the sample (hot side), and heat was conducted through the sample to the cold side. Once thermal equilibrium was established between the hot and cold plates, the steady-state heat flux was measured by a heat-flux sensor, and the thermal conductivity κ was calculated using the following equation:

$$k = \frac{f ed}{\Delta T} \tag{S7}$$

where f is the calibration coefficient of the heat-flux sensor (in W·m^{-2}·mV^{-1}), e is the output voltage of the sensor (mV), d is the sample thickness (m), and ΔT is the temperature difference between the hot and cold sides (K). The detailed measurement procedure is as follows. The instrument was first calibrated using a standard reference sample. Thermal conductivity measurements were then carried out on both polycrystalline and single-crystal samples. The polycrystalline sample was cylindrical with a diameter of 12 mm and a height of 8.4 mm. The single-crystal sample was rectangular with dimensions of 10 mm by 10 mm by 6 mm. Both types of samples were placed into the test chamber under a pressure of 30 N with the cold-side temperature set to 26 °C and the initial hot-side temperature set to 30 °C. After the system reached steady state, the instrument automatically recorded five readings and reported their average. To ensure data reliability, each sample was measured three times at the same temperature and the final thermal conductivity value was taken as the average of these three measurements. In addition to account for heat flux fringing effects caused by the mismatch between sample and sensor areas, the thermal conductivity of air was also measured under identical temperature and thickness conditions and the true thermal conductivity of each sample was corrected using the following area-weighted formula:

$$\frac{Ssample}{Ssensor} \cdot k_{sample} + \frac{Sair}{Ssensor} \cdot k_{air} = k_{test} \tag{S8}$$

For the polycrystalline sample S_{sample}=113.04 mm^2 and for the single-crystal sample S_{sample}=100 mm^2, while S_{sensor}= S_{air}=900 mm^2. Finally, by gradually increasing the hot-side temperature to enlarge the temperature difference, thermal conductivity measurements were carried out at multiple temperatures.

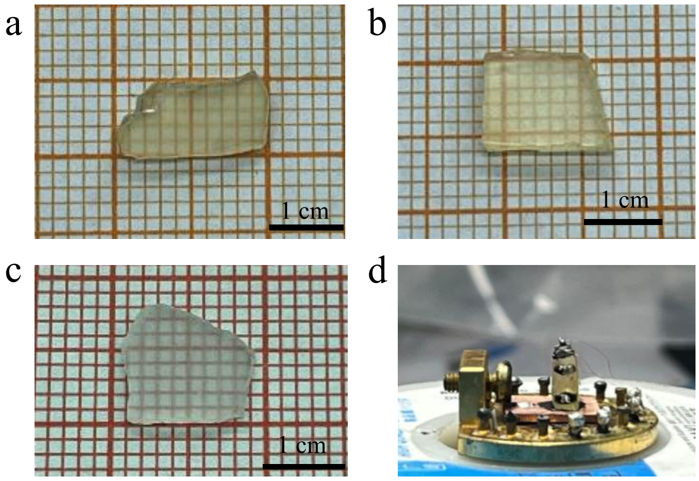

Figure S11. Optical photograph of single-crystal Cs_2ZnI_4 along (a) [100], (b) [010] and (c) [001] orientations for laser flash measurement, and (d) single-crystal Cs_2ZnI_4 along [001] for steady-state measurement.

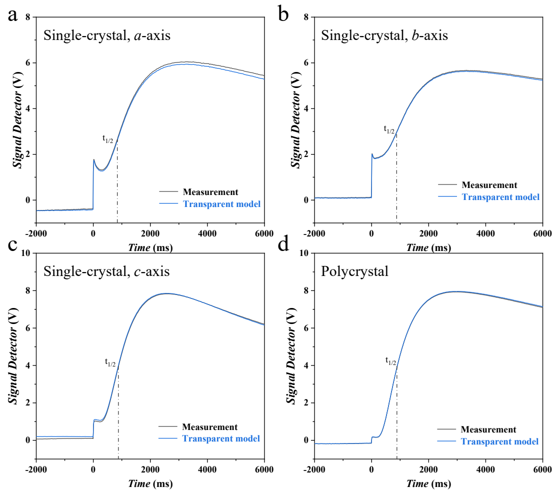

Figure S12. Raw data of thermal diffusivity measurement at 300 K via laser flash method, for single-crystal Cs_2ZnI_4 along (a) [100], (b) [010] and (c) [001] orientations, and for polycrystalline Cs_2ZnI_4 (d).

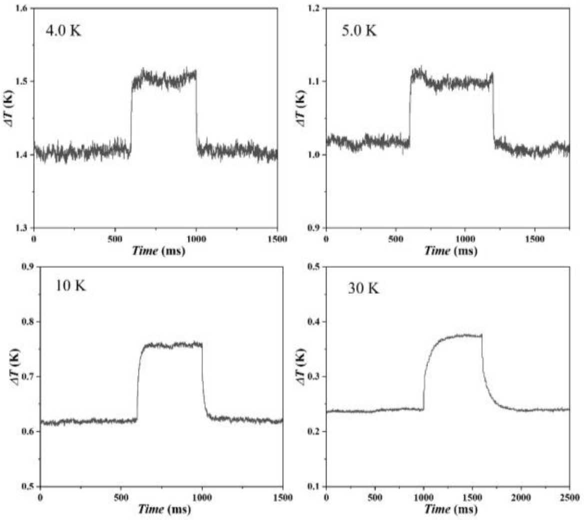

Figure S13. Raw data of thermal conductivity measurement at different temperatures via the steady-state method, for single-crystal Cs_2ZnI_4 [001] orientations.

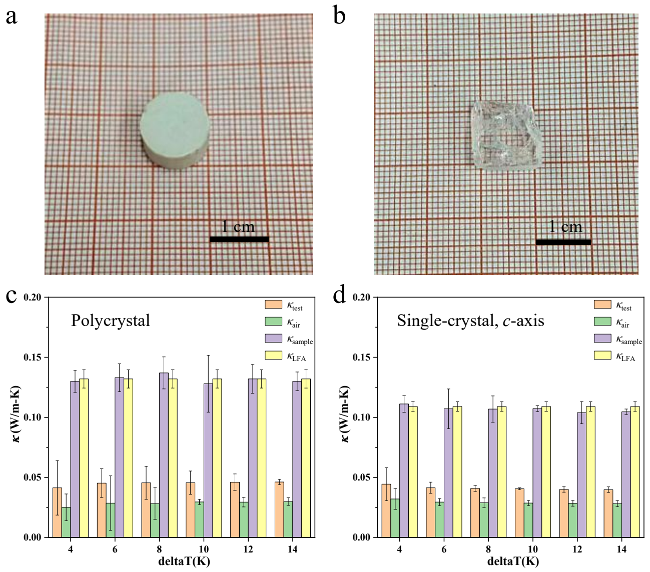

Figure S14. (a-b) Optical images of polycrystalline and single-crystal Cs_2ZnI_4 and (c-d) corresponding thermal conductivity measurements.

Table S5. Measured and corrected thermal conductivity of polycrystal Cs_2ZnI_4 using the guarded-hot-plate method at various ΔT.

T_{hot} (°C)	T_{cold} (°C)	ΔT (K)	κ_{test} (W/(m-K))	κ_{air} (W/(m-K))	κ_{sample} (W/(m-K))	κ_{LFA} (W/(m-K))	Discrepancy (%)
30	26	4	0.04136	0.02509	0.130	0.132	1.5
32	26	6	0.04527	0.02858	0.133	0.132	0.7
34	26	8	0.04551	0.02825	0.137	0.132	5.3
36	26	10	0.04561	0.02958	0.128	0.132	3.0
38	26	12	0.04603	0.02942	0.132	0.132	0
40	26	14	0.04618	0.02991	0.130	0.132	1.5

Table S6. Measured and corrected thermal conductivity of single crystal *c*-axis Cs_2ZnI_4 using the guarded-hot-plate method at various ΔT.

T_{hot} (°C)	T_{cold} (°C)	ΔT (K)	κ_{test} (W/(m-K))	κ_{air} (W/(m-K))	κ_{sample} (W/(m-K))	κ_{LFA} (W/(m-K))	Discrepancy (%)
30	26	4	0.04441	0.03206	0.111	0.109	1.8
32	26	6	0.04138	0.02948	0.107	0.109	1.8
34	26	8	0.04080	0.02892	0.107	0.109	1.8
36	26	10	0.04059	0.02867	0.107	0.109	1.8
38	26	12	0.03997	0.02843	0.104	0.109	4.6
40	26	14	0.03981	0.02819	0.105	0.109	3.7

Table S7. Calculated room-temperature thermal conductivity of Cs_2ZnI_4 based on the phonon Wigner transport equation, including particle-like and wave-like contributions.

	xx	yy	zz	yz	xz	xy
κ_P	0.249	0.257	0.253	0	0	0
κ_C	0.044	0.048	0.043	0	0	0
$\kappa_{TOT}= \kappa_P+\kappa_C$	0.293	0.305	0.296	0	0	0

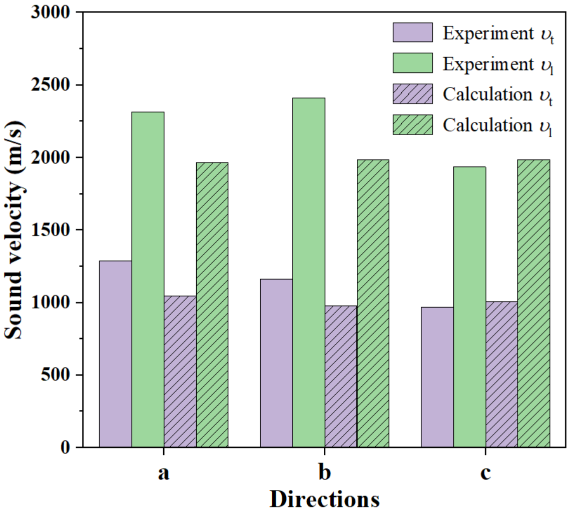

Figure S15. Experimental and calculated longitudinal and transverse sound velocities along the *a*, *b*, and *c* directions of Cs_2ZnI_4 single crystal.

Section S5: Other measurements

The sound velocity was measured at room temperature using an ultrasonic pulse receiver (Olympus-NDT) equipped with an oscilloscope (Keysight). Room temperature Raman spectra were measured in back-scattering with a Jobin-Yvon HR800 Raman system. The instrument was equipped with a liquid nitrogen-cooled CCD detector, a 100× objective lens (numerical aperture, NA= 0.90), and a 1800 lines/mm grating. A diode-pumped-solid-state laser operating at 532 nm excitation wavelength was employed, with plasma lines removed from the laser signals using BragGrate Bandpass filters. Three BragGrate notch filters (OptiGrate Corp.), each exhibiting an optical density of 4 and a full width at half maximum (FWHM) of 5 cm^{-1} [172], enabled measurements down to 5 cm^{-1} for each excitation. The laser power was kept below 200 µW to avoid sample heating. The optical band gap was measured by UV-3600 Plus (Shimadzu). X-ray photoelectron spectroscopy (XPS, Al-K*α* radiation) was conducted with ESCALAB 250Xi (Thermo Fisher Scientific). The ionic conductivity was measured by the Chenhua 760 electrochemical workstation. Infrared photos were taken by HINMICRO K20.

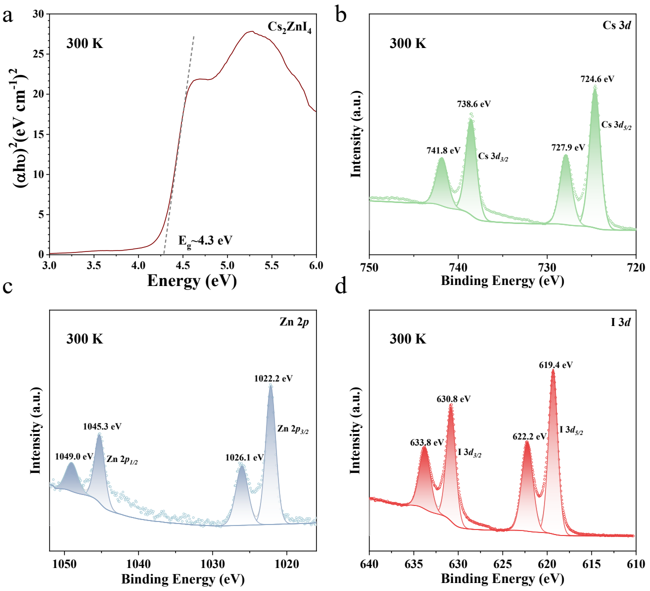

Figure S16. Optical band gap (a), and X-ray photoelectron spectroscopy (b-d) of Cs_2ZnI_4.

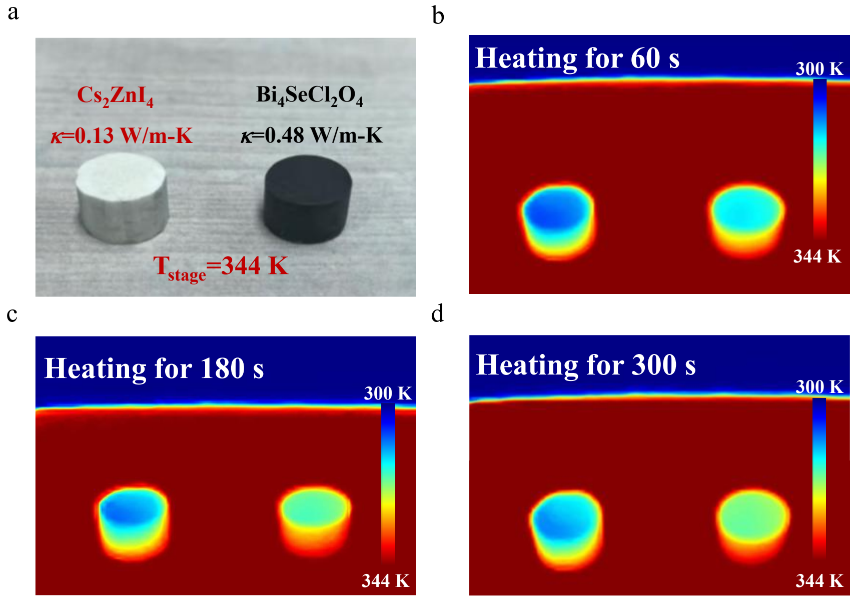

Figure S17. The heat insulation of Cs_2ZnI_4 compared to $Bi_4SeCl_2O_4$[173-175] with the same-sized cylinders placed on a heating stage monitored by optical (a) and infrared cameras observe heating for 60 s (b), 180 s (c) and 300 s (d).

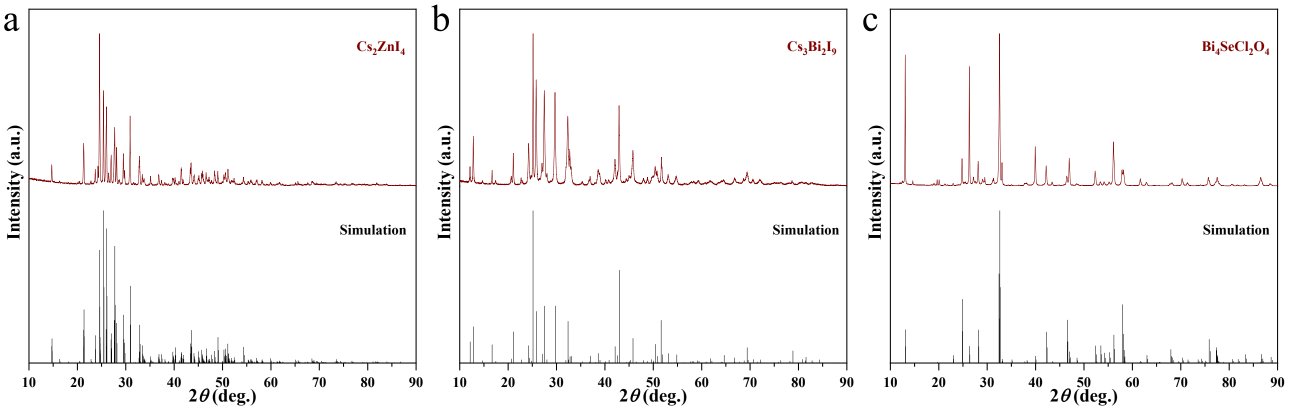

Figure S18. X-ray diffraction (XRD) pattern of cylinders (a) Cs_2ZnI_4, (b) $Cs_3Bi_2I_9$ and (c) $Bi_4SeCl_2O_4$.

Supplementary references

1 Braul, H. & Plint, C. A. Elastic and photoelastic constants of NaCl, KBr and LiF by Brillouin scattering. *Solid State Communications* **38**, 227-230 (1981).
2 Chen, Z. *et al.* Analytical approach to structural chemistry origins of mechanical, acoustical and thermal properties. *National Science Review* **11**, nwae269 (2024).
3 Huntington, H. B. Solid State Physics. 213-351 (1958).
4 Gong, Z. *et al.* Monte Carlo and lattice-dynamics studies of the thermal and elastic properties of a rigid-ion model of sodium chloride. *Physical Review B* **38**, 10820-10829 (1988).
5 Gong, Z. *et al.* van der Waals forces in a Monte Carlo and lattice-dynamics study of the thermal and elastic properties of a rigid-ion model of potassium chloride. *Physical Review B* **40**, 3294-3300 (1989).
6 Goyal, S. C. & Verma, M. P. Dielectric behavior of allkali halides and the second-neighbor interaction. *Physical Review B* **9**, 3609-3613 (1974).
7 Groenewegen, P. P. M. & Huiszoon, C. Debye-Waller B values for some NaCl-type structures and interionic interaction. *Acta Crystallographica Section A* **28**, 166-169 (1972).
8 Chatterjee, S. *et al.* Unified study of the lattice-mechanical properties of copper halide crystals. *Physical Review B* **28**, 3534-3549 (1983).
9 Kocak, B. *et al.* A first-principle study of the structural, elastic, lattice dynamical and thermodynamic properties of PrX (X=P, As). *Physica B: Condensed Matter* **407**, 316-323 (2012).
10 Slagle, O. D. & McKinstry, H. A. Temperature Dependence of the Elastic Constants of the Alkali Halides. III. CsCl, CsBr, and CsI. *Journal of Applied Physics* **38**, 451-458 (1967).
11 Dandekar, D. P. Pressure Dependence of the Elastic Constants of Calcite. *Physical Review* **172**, 873-877 (1968).
12 Kim, K. *et al.* Elastic constants and related properties of tetrahedrally bonded BN, AlN, GaN, and InN. *Physical Review B* **53**, 16310-16326 (1996).
13 Azuhata, T. *et al.* Elastic constants of III - V compound semiconductors: modification of Keyes' relation. *Journal of Physics: Condensed Matter* **8**, 3111 (1996).
14 Pedersen, T. G. & Pedersen, J. G. Self-consistent tight-binding model of B and N doping in graphene. *Physical Review B* **87**, 155433 (2013).
15 Deligoz, E. *et al.* Elastic, electronic, and vibrational properties of RhN compound. *Journal of Materials Science* **45**, 3720-3726 (2010).
16 Balzarotti, A. *et al.* Short-range order and clustering in $Ga_{1-x}Al_xAs$ and its heterostructures. *Solid State Communications* **56**, 471-473 (1985).
17 Lam, P. K. *et al.* Analytic relation between bulk moduli and lattice constants. *Physical Review B* **35**, 9190-9194 (1987).
18 Rimai, D. S. & Sladek, R. J. Elastic moduli and mode gammas of GaP: Their relationship to those of other isomorphic crystals and the high pressure structural-electrical transition. *Solid State Communications* **30**, 591-594 (1979).
19 Di Ventra, M. *et al.* Chemical and structural contributions to the valence-band offset at GaP/GaAs heterojunctions. *Physical Review B* **54**, 5691-5695 (1996).
20 Nichols, D. N. *et al.* Elastic anharmonicity of InP: Its relationship to the high pressure transition. *Solid State Communications* **36**, 667-669 (1980).
21 Carles, R. *et al.* Resonance Raman scattering in InAs near the E1 edge. *Physical Review B* **22**, 6120-6126 (1980).
22 Kobayashi, A. & Roy, A. Effects of local atomic arrangements on the densities of phonon states of $Ga_{1-x}In_xAs$, $Ga_{1-x}In_xSb$, $GaAs_{1-x}Sb_x$, and $InAs_{1-x}Sb_x$. *Physical Review B* **35**, 5611-5620 (1987).
23 BENTLE, G. G. Elastic Constants of Single-Crystal BeO at Room Temperature. *Journal of the American Ceramic Society* **49**, 125-128 (1966).
24 Jog *et al.* Three-body-interaction effects on the phase-transition and high-pressure behavior of divalent-metal oxides. *Physical review. B, Condensed matter* **35 10**, 5235-5243 (1986).
25 Dobrzyniecki, J. *et al.* Effective three-body interactions for bosons in a double-well confinement. *Physical Review A* **97**, 013609 (2018).
26 Peselnick, L. & Meister, R. L. Variational Method of Determining Effective Moduli of Polycrystals: (A) Hexagonal Symmetry, (B) Trigonal Symmetry. *Journal of Applied Physics* **36**, 2879-2884 (1965).
27 Vogelgesang, R. *et al.* The elastic constants of single crystal β-Si_3N_4. *Applied Physics Letters* **76**, 982-984 (2000).
28 Oliinyk, O. V. & Tatarenko, V. A. Modulated distribution of vacancies within the post-irradiated bcc metals. *Low Temperature Physics* **49**, 1229-1235 (2023).
29 Rajput, B. & Browne, D. A. Lattice dynamics of II-VI materials using the adiabatic bond-charge model. *Physical review. B, Condensed matter* **53 14**, 9052-9058 (1995).
30 Peng, F. *et al.* First-principles calculations on phase transition and elasticity of CdO under pressure. *Solid State Communications* **148**, 6-9 (2008).
31 Deligoz, E. *et al.* Elastic, electronic, and lattice dynamical properties of CdS, CdSe, and CdTe. *Physica B-condensed Matter* **373**, 124-130 (2006).
32 Prieur, J. Y. *et al.* Elastic Constants of β-HgS. *Acta Physica Polonica A* **94**, 487-491 (1998).
33 Oliver, D. W. The Elastic Moduli of MnO. *Journal of Applied Physics* **40**, 893-893 (1969).
34 Herrmann-Ronzaud, D. *et al.* Critical and elastic behaviour of paramagnetic manganese oxide. *Physica B+C* **86-88**, 570-571 (1977).
35 in *Project T. M.* (United States: N. p., 2020).
36 in *Project, T. M.* (United States: N. p., 2020).
37 Gupta, B. R. K. & Kumar, V. Analysis of effective compressibilities in PbS, PbSe, PbTe and SnTe. *Solid State Communications* **45**, 745-747 (1983).
38 Skelton, J. M. *et al.* Thermal physics of the lead chalcogenides PbS, PbSe, and PbTe from first principles. *Physical Review B* **89**, 205203 (2014).
39 Hsueh, H. C. *et al.* High-pressure effects in the layered semiconductor germanium selenide. *Phys Rev B Condens Matter* **51**, 16750-16760 (1995).
40 Rabe, K. M. & Joannopoulos, J. D. Structural properties of GeTe at T=0. *Physical Review B* **36**, 3319-3324 (1987).
41 in *Project, T. M.* (United States: N. p., 2020).
42 Rodríguez-Hernández, P. *et al.* Electronic and structural properties of cubic BN and BP. *Physical Review B* **51**, 14705-14708 (1995).
43 Cline, C. F. *et al.* Elastic Constants of Hexagonal BeO, ZnS, and CdSe. *Journal of Applied Physics* **38**, 1944-1948 (1967).
44 Xia, H. *et al.* BP at megabar pressures and its equation of state to 110 GPa. *Journal of Applied Physics* **74**, 1660-1662 (1993).
45 Broido, D. A. *et al.* *Ab initio* study of the unusual thermal transport properties of boron arsenide and related materials. *Physical Review. B, Condensed Matter and Materials Physics* **88**, Medium: X; Size: p. 144306 (2013).
46 Gerlich, D. *et al.* Elastic properties of aluminum nitride. *Journal of Physics and Chemistry of Solids* **47**, 437-441 (1986).
47 Adachi, S. GaAs, AlAs, and $Al_xGa_{1-x}As$: Material parameters for use in research and device applications. *Journal of Applied Physics* **58**, R1-R29 (1985).
48 Bolef, D. I. & Menes, M. Elastic Constants of Single-Crystal Aluminum Antimonide. *Journal of Applied Physics* **31**, 1426-1427 (1960).
49 Yamaguchi, M. *et al.* Brillouin scattering study of gallium nitride: elastic stiffness constants. *Journal of Physics: Condensed Matter* **9**, 241 (1997).
50 Weil, R. & Groves, W. O. The Elastic Constants of Gallium Phosphide. *Journal of Applied Physics* **39**, 4049-4051 (1968).
51 Blakemore, J. S. Semiconducting and other major properties of gallium arsenide. *Journal of Applied Physics* **53**, R123-R181 (1982).
52 Brazhkin, V. V. *et al.* Elastic softness of amorphous tetrahedrally bonded GaSb and $(Ge_2)_{0.27}(GaSb)_{0.73}$ semiconductors. *Physical Review B* **56**, 990-993 (1997).
53 Every, A. G. *et al.* Optimized determination of elastic constants of crystals and their uncertainties from surface Brillouin scattering. *Ultrasonics* **69**, 273-278 (2016).
54 Hickernell, F. S. & Gayton, W. Elastic Constants of Single-Crystal Indium Phosphide. *Journal of Applied Physics* **37**, 462-462 (1966).
55 Gerlich, D. Elastic Constants of Single-Crystal Indium Arsenide. *Journal of Applied Physics* **34**, 2915-2915 (1963).
56 Drabble, J. R. & Brammer, A. J. The third-order elastic constants of indium antimonide. *Proceedings of the Physical Society* **91**, 959 (1967).
57 Sumino, Y. *et al.* MEASUREMENT OF ELASTIC CONSTANTS AND INTERNAL FRICTIONS ON SINGLE-CRYSTAL MgO BY RECTANGULAR PARALLELEPIPED RESONANCE. *Journal of Physics of the Earth* **24**, 263-273 (1976).
58 Chang, Z. P. & Graham, E. K. Elastic properties of oxides in the NaCl-structure. *Journal of Physics and Chemistry of Solids* **38**, 1355-1362 (1977).
59 Carlotti, G. *et al.* Brillouin scattering determination of the whole set of elastic constants of a single transparent film of hexagonal symmetry. *Journal of Physics: Condensed Matter* **7**, 9147 (1995).
60 Lee, B. H. Elastic Constants of ZnTe and ZnSe between 77°–300°K. *Journal of Applied Physics* **41**, 2984-2987 (1970).
61 Zarembovitch, A. Étude des constantes élastiques de la blende et de leur variation avec la température. *Journal De Physique* **24**, 1097-1102 (1963).
62 Noguera, A. & Wasim, S. M. Lattice thermal conductivity of II–VI

compounds. *Solid State Communications* **50**, 483-486 (1984).
63 Subhadra, K. G. & Sirdeshmukh, D. B. X-ray determination of the mean Debye-Waller factors, amplitudes of vibrations and the Debye temperatures of CdO, PbS and MnS. *Pramana* **10**, 357-360 (1978).
64 Holland, M. G. Phonon Scattering in Semiconductors From Thermal Conductivity Studies. *Physical Review* **134**, A471-A480 (1964).
65 Bonello, B. & Fernandez, B. Elastic constants of CdSe at low temperature. *Journal of Physics and Chemistry of Solids* **54**, 209-212 (1993).
66 Kumazaki, K. Elastic properties and ionicity of zero-gap semiconductors. *Physica Status Solidi (a)* **33**, 615-623 (1976).
67 Ford, P. J. *et al.* The effects of pressure on the elastic constants of mercury selenide up to the phase transition. *Journal of Physics C: Solid State Physics* **15**, 657 (1982).
68 Uchida, N. & Saito, S. Elastic Constants and Acoustic Absorption Coefficients in MnO, CoO, and NiO Single Crystals at Room Temperature. *Journal of the Acoustical Society of America* **51**, 1602-1605 (1972).
69 Vekilov, Y. K. R., A. ELASTIC CONSTANTS AND LATTICE DYNAMICS OF SOME A 2 B 6 COMPOUNDS. *Soviet Physics—Solid State [translation of Fizika Tverdogo Tela (Leningrad)]* **13**, 956-960 (1971).
70 Xu, Y. *et al.* Performance optimization and single parabolic band behavior of thermoelectric MnTe. *Journal of Materials Chemistry A* **5**, 19143-19150 (2017).
71 Ding, G. *et al.* High-efficient thermoelectric materials: The case of orthorhombic IV-VI compounds. *Scientific Reports* **5**, 9567 (2015).
72 Li, W. *et al.* Band and scattering tuning for high performance thermoelectric $Sn_{1-x}Mn_xTe$ alloys. *Journal of Materiomics* **1**, 307-315 (2015).
73 Wang, H. *et al.* High Thermoelectric Efficiency of n-type PbS. *Advanced Energy Materials* **3**, 488-495 (2013).
74 Chen, Z. *et al.* Vacancy-induced dislocations within grains for high-performance PbSe thermoelectrics. *Nature Communications* **8**, 13828 (2017).
75 Zhang, X. *et al.* Thermoelectric properties of GeSe. *Journal of Materiomics* **2**, 331-337 (2016).
76 Zhang, X. *et al.* GeTe Thermoelectrics. *Joule* **4**, 986-1003 (2020).
77 Prevot, B. *et al.* Phonon spectrum of CuCl at 4.2K and its temperature dependence. *Journal of Physics C: Solid State Physics* **10**, 3999-4011 (1977).
78 Hanson, R. C. *et al.* Elastic and piezoelectric constants of the cuprous halides. *Applied Physics Letters* **21**, 490-492 (1972).
79 Hughes, W. C. & Cain, L. S. Second-order elastic constants of AgCl from 20 to 430°C. *Physical Review B* **53**, 5174-5180 (1996).
80 Dorner, B. *et al.* Lattice dynamics of AgBr. *Journal of Physics C: Solid State Physics* **9**, 723-732 (1976).
81 San-Guo, S. Calculation of the elastic properties of semiconductors. *Journal of Physics: Condensed Matter* **6**, 8733 (1994).
82 Kodama, M. *et al.* Pressure Dependence of the Elastic Constants of TlCl. *Journal of the Physical Society of Japan* **33**, 1361-1371 (1972).
83 Morse, G. E. & Lawson, A. W. The temperature and pressure dependence of the elastic constants of thallium bromide ag. *Journal of Physics and Chemistry of Solids* **28**, 939-950 (1967).
84 Bjerg, L. *et al.* Modeling the thermal conductivities of the zinc antimonides ZnSb and Zn_4Sb_3. *Physical Review B* **89**, 024304 (2014).
85 Madelung, O. Semiconductors: Data Handbook. 476-506 (Springer, 2004).
86 D. B. Sirdeshmukh, L. S., Subhadra, K. G. Alkali Halides. (Springer Berlin).
87 Tsay, Y.-f. *et al.* Theory of the Temperature Derivative of the Refractive Index in Transparent Crystals. *Physical Review B* **8**, 2688-2696 (1973).
88 Boyer, L. L. First-principles equation-of-state calculations for alkali halides. *Physical Review B* **23**, 3673-3685 (1981).
89 Berger, L. I. Semiconductor Materials. (CRC Press, 1996).
90 Abendroth, B. *et al.* X-ray diffraction study of stress relaxation in cubic boron nitride films grown with simultaneous medium-energy ion bombardment. *Applied Physics Letters* **85**, 5905-5907 (2004).
91 Slack, G. A. & Bartram, S. F. Thermal expansion of some diamondlike crystals. *Journal of Applied Physics* **46**, 89-98 (1975).
92 Benkabou, F. *et al.* Atomistic study of zinc-blende BAs from molecular dynamics. *Physics Letters A* **252**, 71-76 (1999).
93 Magri, R. & Zunger, A. Effects of interfacial atomic segregation and intermixing on the electronic properties of InAs/GaSb superlattices. *Physical Review B* **65**, 165302 (2002).
94 Lu, L.-Y. *et al.* Thermodynamic properties of MgO under high pressure from first-principles calculations. *Physica B: Condensed Matter* **370**, 236-242 (2005).
95 Fischer, W. A. & Janke, D. Electrolytic deoxidation of liquid metals at 1600°C. *Scripta Metallurgica* **6**, 923-928 (1972).
96 Beals, R. J. & Cook, R. L. Directional Dilatation of Crystal Lattices at Elevated Temperatures. *Journal of the American Ceramic Society* **40**, 279-284 (1957).
97 Zollweg, R. J. X-Ray Lattice Constant of Barium Oxide. *Physical Review* **100**, 671-673 (1955).
98 Singh, H. P. & Dayal, B. Lattice parameters of cadmium oxide at elevated temperatures. *Solid State Communications* **7**, 725-726 (1969).
99 Kuriyama, M. & Hosoya, S. X-ray Measurement of Scattering Factors of Manganese and Oxygen Atoms in Manganous Oxide. *Journal of the Physical Society of Japan* **17**, 1022-1029 (1962).
100 Zhang, Y. *et al.* Microstructures and properties of high-entropy alloys. *Progress in Materials Science* **61**, 1-93 (2014).
101 Sternberg, Y. *et al.* Lattice matching and thermal expansion in the Pb-Sn-Te system. *Journal of Solid State Chemistry* **43**, 364-367 (1982).
102 Smith, T. F. & White, G. K. The low-temperature thermal expansion and Gruneisen parameters of some tetrahedrally bonded solids. *Journal of Physics C: Solid State Physics* **8**, 2031 (1975).
103 Sauder, T. *et al.* Effects of uniaxial stress on the excitons in single crystals of CuI: Comparison with thin films. *Solid State Communications* **51**, 323-326 (1984).
104 Tallon, J. L. & Buckley, R. G. The fast-ion transition in FCC silver iodide. *Solid State Communications* **47**, 563-566 (1983).
105 Pathak, P. D. & Vasavada, N. G. Thermal expansion of LiF by X-ray diffraction and the temperature variation of its frequency spectrum. *Acta Crystallographica Section A* **28**, 30-33 (1972).
106 Yagi, T. Experimental determination of thermal expansivity of several alkali halides at high pressures. *Journal of Physics and Chemistry of Solids* **39**, 563-571 (1978).
107 Rapp, J. E. & Merchant, H. D. Thermal expansion of alkali halides from 70 to 570 K. *Journal of Applied Physics* **44**, 3919-3923 (1973).
108 Wang, K. & Reeber, R. R. High temperature thermal expansion of alkali halides. *Journal of Physics and Chemistry of Solids* **56**, 895-900 (1995).
109 Bailey, A. C. & Yates, B. The low temperature thermal expansion and related thermodynamic properties of alkali halides with a caesium chloride structure. *Philosophical Magazine* **16**, 1241-1248 (1967).
110 Johnson, J. W. *et al.* Molar Volume and Structure of Solid and Molten Cesium Halides. *Journal of the American Chemical Society* **77**, 2734-2737 (1955).
111 Colella, R. Reply to Post's comments on my paper Multiple diffraction of X-rays and the phase problem. Computational procedures and comparison with experiment. *Acta Crystallographica Section A* **31**, 155-155 (1975).
112 Kittel, C. Introduction to Solid State Physics. (John Wiley & Sons, 2005).
113 Phillips, J. C. Ionicity of the Chemical Bond in Crystals. *Reviews of Modern Physics* **42**, 317-356 (1970).
114 Chen, Z. *et al.* Rationalizing phonon dispersion for lattice thermal conductivity of solids. *National Science Review* **5**, 888-894 (2018).
115 Morelli D T & A, S. G. High lattice thermal conductivity solids. (Springer New York, 2006).
116 Steigmeier, E. F. & Kudman, I. Acoustical-Optical Phonon Scattering in Ge, Si, and III-V Compounds. *Physical Review* **141**, 767-774 (1966).
117 Spitzer, D. P. Lattice thermal conductivity of semiconductors: A chemical bond approach. *Journal of Physics and Chemistry of Solids* **31**, 19-40 (1970).
118 Krukowski, S. *et al.* Thermal properties of indium nitride. *Journal of Physics and Chemistry of Solids* **59**, 289-295 (1998).
119 Slack, G. A. & Newman, R. Thermal Conductivity of MnO and NiO. *Physical Review Letters* **1**, 359-360 (1958).
120 Lewis, F. B. & Saunders, N. H. The thermal conductivity of NiO and CoO at the Neel temperature. *Journal of Physics C: Solid State Physics* **6**, 2525 (1973).
121 Ren, Y. *et al.* Synergistic effect by Na doping and S substitution for high thermoelectric performance of p-type MnTe. *Journal of Materials Chemistry C* **5**, 5076-5082 (2017).
122 Okhotin, A. S. *et al.* Thermal Conductivity of GeS and GeSe. *physica status solidi (b)* **31**, 485-487 (1969).
123 Fernelius, N. C. Properties of gallium selenide single crystal. *Progress in Crystal Growth and Characterization of Materials* **28**, 275-353 (1994).
124 Anis-ur-Rehman, M. & Maqsood, A. Measurement of Thermal Transport Properties with an Improved Transient Plane Source Technique. *International Journal of Thermophysics* **24**, 867-883 (2003).
125 Hakansson, B. & Ross, R. G. Thermal conductivity and heat capacity of solid LiBr and RbF under pressure. *Journal of Physics: Condensed Matter* **1**, 3977 (1989).

126 Ross, R. G. *et al.* Thermal conductivity of solids and liquids under pressure. *Reports on Progress in Physics* **47**, 1347 (1984).
127 Andersson, P. Thermal conductivity under pressure and through phase transitions in solid alkali halides. I. Experimental results for KCl, KBr, KI, RbCl, RbBr and RbI. *Journal of Physics C: Solid State Physics* **18**, 3943 (1985).
128 Gerlich, D. & Andersson, P. Temperature and pressure effects on the thermal conductivity and heat capacity of CsCl, CsBr and CsI. *Journal of Physics C: Solid State Physics* **15**, 5211 (1982).
129 Mandal, S. & Sarkar, P. Computational Exploration of Ultralow Lattice Thermal Conductivity and High Figure of Merit in p-Type Bulk RbX_2Sb (X = K, Na). *ACS Applied Energy Materials* **6**, 939-949 (2023).
130 Li, W. *et al.* Crystal Structure Induced Ultralow Lattice Thermal Conductivity in Thermoelectric Ag_9AlSe_6. *Advanced Energy Materials* **8**, 1800030 (2018).
131 Jin, M. *et al.* Fabrication and Thermoelectric Properties of Single-Crystal Argyrodite Ag_8SnSe_6. *Chemistry of Materials* **31**, 2603-2610 (2019).
132 Lin, S. *et al.* High Thermoelectric Performance of Ag_9GaSe_6 Enabled by Low Cutoff Frequency of Acoustic Phonons. *Joule* **1**, 816-830 (2017).
133 Cherniushok, O. *et al.* Lone-Pair-Like Interaction and Bonding Inhomogeneity Induce Ultralow Lattice Thermal Conductivity in Filled β-Manganese-Type Phases. *Chemistry of Materials* **34**, 6389-6401 (2022).
134 Jiang, L. *et al.* Cu_2Se as Textured Adjuvant for Pb-Doped BiCuSeO Materials Leading to High Thermoelectric Performance. *ACS Applied Materials and Interfaces* **13**, 11977-11984 (2021).
135 Xiao, Y. *et al.* Origin of low thermal conductivity in SnSe. *Physical Review B* **94**, 125203 (2016).
136 Kurosaki, K. *et al.* Ag_9TlTe_5: A high-performance thermoelectric bulk material with extremely low thermal conductivity. *Applied Physics Letters* **87** (2005).
137 Pei, Y. *et al.* Multiple Converged Conduction Bands in $K_2Bi_8Se_{13}$: A Promising Thermoelectric Material with Extremely Low Thermal Conductivity. *Journal of the American Chemical Society* **138**, 16364-16371 (2016).
138 Zhang, X. *et al.* Promising Thermoelectric $Ag_{5-\delta}Te_3$ with Intrinsic Low Lattice Thermal Conductivity. *ACS Energy Letters* **2**, 2470-2477 (2017).
139 Das, A. *et al.* Strong Antibonding I (p)-Cu (d) States Lead to Intrinsically Low Thermal Conductivity in $CuBiI_4$. *Journal of the American Chemical Society* **145**, 1349-1358 (2023).
140 Acharyya, P. *et al.* Extended Antibonding States and Phonon Localization Induce Ultralow Thermal Conductivity in Low Dimensional Metal Halide. *Advanced Functional Materials* **33**, 2304607 (2023).
141 Rahman Rano, B. *et al.* Elastic, electronic, bonding, and optical properties of WTe_2 Weyl semimetal: A comparative investigation with $MoTe_2$ from first principles. *Results in Physics* **19**, 103639 (2020).
142 Rano, B. U. R. *et al.* Ab initio approach to the elastic, electronic, and optical properties of $MoTe_2$ topological Weyl semimetal. *Journal of Alloys and Compounds* (2019).
143 Lagnier, R. *et al.* Specific heat of the semiconducting layered compound $SnSe_2$ at low temperatures. *Solid State Communications* **48**, 65-68 (1983).
144 Li, C. W. *et al.* Orbitally driven giant phonon anharmonicity in SnSe. *Nature Physics* **11**, 1063-1069 (2015).
145 Madelung, O. in Semiconductors: Data Handbook. (Springer, 2004).
146 Powell, B. M. *et al.* Anisotropic phonon dispersion in GaS. *Journal of Physics C: Solid State Physics* **10**, 3039 (1977).
147 Khalilov K M, R. K. I. PREPARATION OF A GASE SINGLE CRYSTAL AND DETERMINATION OF ITS ELASTIC PARAMETERS. *Soviet Physics Crystallography* **Ussr 11**, 786 (1967).
148 Bhan, S. & Schubert, K. Kristallstruktur von Tl_5Te_3 und Tl_2Te_3. *Journal of the Less Common Metals* **20**, 229-235 (1970).
149 Gatulle, M. *et al.* Elastic constants of the layered compounds Gas, GaSe, InSe, and their pressure dependence I. Experimental part. *physica status solidi (b)* **119**, 327-336 (1983).
150 Jana, M. K. *et al.* The Origin of Ultralow Thermal Conductivity in InTe: Lone-Pair-Induced Anharmonic Rattling. *Angewandte Chemie International Edition* **55**, 7792-7796 (2016).
151 Every, A. M., A. 593-606 (Springer).
152 Yang, F. *et al.* Effective thermal conductivity of polycrystalline materials with randomly oriented superlattice grains. *Journal of Applied Physics* **108**, 034310-034310 (2010).
153 Kullmann, W. *et al.* Lattice Dynamics and Phonon Dispersion in the Narrow Gap Semiconductor Bi_2Te_3 with Sandwich Structure. *physica status solidi (b)* **162**, 125-140 (1990).
154 Sheldrick, G. SHELXT - Integrated space-group and crystal-structure determination. *Acta Crystallographica Section A* **71**, 3-8 (2015).
155 Dolomanov, O. V. *et al.* OLEX2: a complete structure solution, refinement and analysis program. *Journal of Applied Crystallography* **42**, 339-341 (2009).
156 Guy, J. in https://sourceforge.net/projects/cctw/.
157 Osborn, R. W., J. NeXpy. in https://github.com/nexpy/nexpy.
158 Osborn, R., Krogstad, M. & Wozniak, J. NXrefine. in https://github.com/rayosborn/nxrefine.
159 Togo, A. First-principles Phonon Calculations with Phonopy and Phono3py. *Journal of the Physical Society of Japan* **92**, 012001 (2022).
160 Hellman, O. *et al.* Temperature dependent effective potential method for accurate free energy calculations of solids. *Physical Review B* **87**, 104111 (2013).
161 Blöchl, P. E. Projector augmented-wave method. *Physical Review B* **50**, 17953-17979 (1994).
162 Perdew, J. P. *et al.* Restoring the Density-Gradient Expansion for Exchange in Solids and Surfaces. *Physical Review Letters* **100**, 136406 (2008).
163 Kresse, G. & Furthmüller, J. Efficient iterative schemes for ab initio total-energy calculations using a plane-wave basis set. *Physical Review B* **54**, 11169-11186 (1996).
164 Eriksson, F. *et al.* The Hiphive Package for the Extraction of High-Order Force Constants by Machine Learning. *Advanced Theory and Simulations* **2**, 1800184 (2019).
165 Silvi, B. & Savin, A. Classification of chemical bonds based on topological analysis of electron localization functions. *Nature* **371**, 683-686 (1994).
166 Maintz, S. *et al.* LOBSTER: A tool to extract chemical bonding from plane-wave based DFT. *Journal of Computational Chemistry* **37**, 1030-1035 (2016).
167 Simoncelli, M. *et al.* Unified theory of thermal transport in crystals and glasses. *Nature Physics* **15**, 809-813 (2019).
168 Momma, K. & Izumi, F. VESTA 3 for three-dimensional visualization of crystal, volumetric and morphology data. *Journal of Applied Crystallography* **44**, 1272-1276 (2011).
169 Stukowski, A. Visualization and analysis of atomistic simulation data with OVITO-the Open Visualization Tool. *Modelling Simul. Mater. Sci. Eng.* **18**, 015012 (2010).
170 Batatia, I. *et al.* A foundation model for atomistic materials chemistry. *The Journal of Chemical Physics* **163** (2025).
171 Plimpton, S. Fast Parallel Algorithms for Short-Range Molecular Dynamics. *Journal of Computational Physics* **117**, 1-19 (1995).
172 Tan, P. H. *et al.* The shear mode of multilayer graphene. *Nature Materials* **11**, 294-300 (2012).
173 Wu, L. *et al.* Thermal transport properties of polycrystalline $Bi_4SeCl_2O_4$ with various texturizations and densities. *Materials Today Physics* **50**, 101618 (2025).
174 Gibson, Q. D. *et al.* Low thermal conductivity in a modular inorganic material with bonding anisotropy and mismatch. *Science* **373**, 1017-1022 (2021).
175 Ji, R. *et al.* Multiple Anion Chemistry for Ionic Layer Thickness Tailoring in $Bi_{2+2n}O_{2+2n}Se_nX_2$ (X = Cl, Br) van der Waals Semiconductors with Low Thermal Conductivities. *Chemistry of Materials* **34**, 4751-4764 (2022).
